# Human cortico-vascular assembloids reveal a CELF2-AHNAK-dependent switch from neuronal to endothelial tropism in glioblastoma cells

**DOI:** 10.1038/s41419-026-09169-z

**Published:** 2026-08-05

**Authors:** Michele Bertacchi, Annie Ladoux, Alexa Saliou, Gwendoline Maharaux, Clément Peux, Laurent Turchi, Béatrice Polo, Hervé Chneiweiss, Elias El-Habr, Marie Pierre Junier, Fabien Almairac, Fanny Burel-Vandenbos, Michèle Studer, Thierry Virolle

**Affiliations:** 1 Université Côte D’Azur, CNRS, Inserm, iBV, Nice, France; 2 DRCI, CHU de Nice, Nice, France; 3 Sorbonne Université, CNRS, INSERM, Institut de Biologie Paris Seine, Center for Neuroscience at Sorbonne Université, 7 Quai Saint-Bernard, Paris, France; 4 Service de Neurochirurgie, Hôpital Pasteur, CHU de Nice, Nice, France; 5 Service d’Anatomopathologie, Hôpital Pasteur, CHU de Nice, Nice, France

**Keywords:** Cancer stem cells, CNS cancer

## Abstract

Glioblastoma (GBM) remains one of the most aggressive human cancers, driven by cellular plasticity and dynamic interactions with the neurovascular niche. Yet, further refinement of existing preclinical models is needed to improve the reproduction of the human brain–vascular interface and facilitate the study of tumor-host crosstalk and invasion. To address this limitation, we developed a human induced pluripotent stem cell (hiPSC)-derived cortico-endothelial (CO + EO) assembloid model by fusing cortical and endothelial organoids. These assembloids spontaneously form branched vascular networks enriched in tight junction proteins (CLDN5, OCLN, ZO-1), thereby recapitulating key BBB-like features and providing a physiologically relevant human platform to investigate GBM–neurovascular interactions. Using this system, we uncovered a previously unrecognized CELF2-dependent glioma stem cell (GSC) tropism. CELF2-expressing GSCs preferentially infiltrate neural regions, where they induce neuronal apoptosis and disrupt endothelial tight junction structure, supporting an aggressive phenotype. In contrast, CELF2-deficient GSCs lose neurotropism, acquire mesenchymal features, and are redirected toward vascular compartments. This endothelial affinity depends on the scaffold protein AHNAK, strongly expressed at the plasma membrane of CELF2-deficient cells and enriched at tumor-endothelial interfaces. AHNAK knockdown partially abolishes endothelial infiltration, demonstrating its role in GBM vascular tropism. Moreover, analysis of patient GBM specimens confirmed that CELF2-positive tumor cells are enriched in poorly vascularized, mitotically active regions and are largely excluded from vessel-rich zones, closely resembling the behavior observed in assembloids. Transcriptomic profiling further revealed that CELF2 promotes a neuronal progenitor-like program while repressing mesenchymal and vascular-associated gene signatures, thereby shaping tumor identity, invasive behavior, and tissue preference. Collectively, this study establishes CO + EO assembloids as an original human model of GBM plasticity at the neurovascular interface. We identify CELF2 as a master regulator of GSC tropism and AHNAK as a mediator of vascular affinity, revealing a molecular axis that governs GBM invasion and providing potential avenues for future therapeutic intervention.

**Figure:**
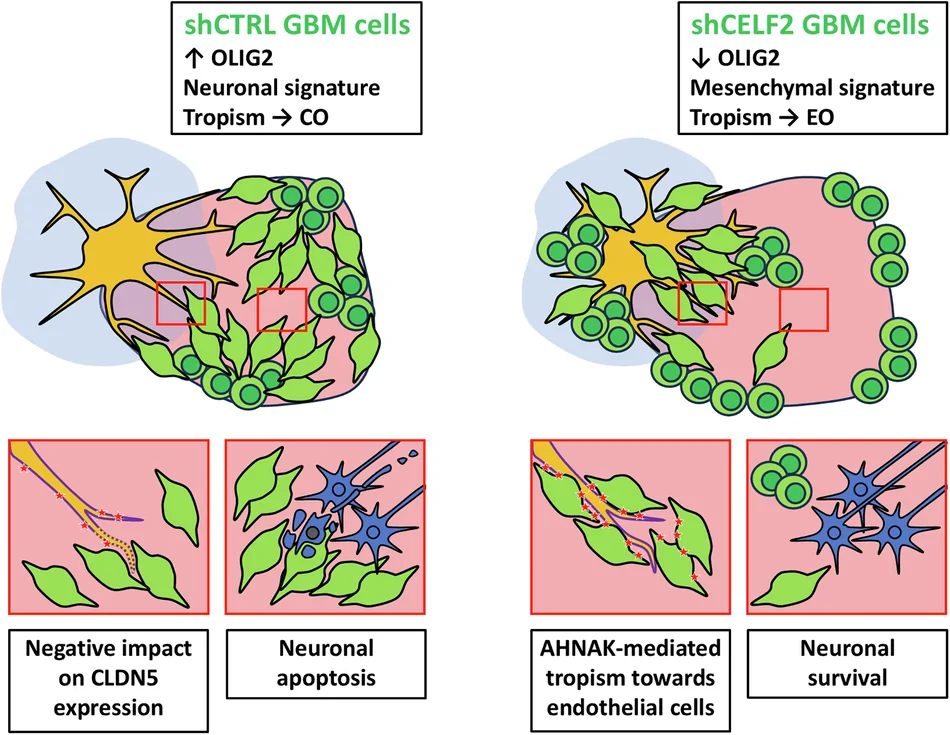
Schematic representation of CO + EO assembloids comprising an endothelial (yellow) and a cortical (red) domain. On the left, control CELF2-expressing GBM cells preferentially infiltrate the neuronal compartment, where they trigger neuronal apoptosis and disrupt the expression of the tight junction protein CLDN5 upon contact with endothelial cells. On the right, CELF2-deprived GBM cells exhibit reduced neural invasion and increased tropism for endothelial cells, consistent with a mesenchymal signature shift partially driven by elevated AHNAK expression. These cells exert limited impact on neuronal viability and BBB integrity, and display partial loss of GSC identity, as indicated by reduced OLIG2 expression. The CO + EO assembloid model thus recapitulates key features of cortico-endothelial human tissue heterogeneity and provides a reliable platform to dissect the molecular mechanisms underlying GSC tropism and their tissue-specific interactions.

Schematic representation of CO + EO assembloids comprising an endothelial (yellow) and a cortical (red) domain. On the left, control CELF2-expressing GBM cells preferentially infiltrate the neuronal compartment, where they trigger neuronal apoptosis and disrupt the expression of the tight junction protein CLDN5 upon contact with endothelial cells. On the right, CELF2-deprived GBM cells exhibit reduced neural invasion and increased tropism for endothelial cells, consistent with a mesenchymal signature shift partially driven by elevated AHNAK expression. These cells exert limited impact on neuronal viability and BBB integrity, and display partial loss of GSC identity, as indicated by reduced OLIG2 expression. The CO + EO assembloid model thus recapitulates key features of cortico-endothelial human tissue heterogeneity and provides a reliable platform to dissect the molecular mechanisms underlying GSC tropism and their tissue-specific interactions.

## Introduction

Glioblastoma (GBM), currently defined as IDH-wild-type, CNS WHO grade 4, is the most common malignant primary brain tumor in adults and represents a major public health challenge due to its highly aggressive nature and poor survival outcomes [1]. Current standard treatments [2] offer only limited improvements in patient life expectancy, largely due to the persistence of treatment-resistant glioma stem cells (GSCs), which drive tumor recurrence and progression through their aggressive and resilient characteristics [3, 4]. In close interaction with the tumor microenvironment, GBM cells display a high degree of plasticity, enabling reversible transitions between an aggressive proliferative GSC state, characterized by the expression of stem cell markers such as NANOG, SOX2, and OLIG2, and a more differentiated, quiescent, and less aggressive phenotype marked by GFAP expression [5, 6]. This dynamic plasticity underlies the functional hierarchy and intra-tumoral heterogeneity that define GBM pathophysiology. Advancing our understanding of key GBM cell behaviors, including proliferation, infiltration, tropism, and crosstalk with the surrounding normal brain tissue, is critical for identifying new therapeutic targets and developing more effective therapeutic strategies.

A hallmark of GBM is its characteristic architecture, consisting of a densely cellular tumor core, often containing hypoxic and necrotic regions with increased vascularization, and an infiltrative tumor periphery, each exhibiting distinct transcriptomic signatures [7, 8]. The tumor core typically comprises a heterogenous mixture of GSCs and more differentiated tumor cells, which may either intermingle or segregate into discrete micro-territories exhibiting variable proliferative activity [6, 9, 10]. The emergence of these micro-territories likely results from continuous communication both among tumor cells and between tumor cells and non-neoplastic brain-resident cell types, which are present in relatively low abundance within the tumor mass. A central component shaping the tumor core is the microvasculature [7]. The newly formed blood vessels are frequently dysfunctional and prone to thrombosis, contributing to hypoxic conditions and the emergence of necrotic areas [7]. Concomitantly, the integrity of the blood–brain barrier (BBB), normally maintained by tightly connected endothelial cells, is compromised, allowing abnormal immune cell infiltration and fostering a pro-inflammatory microenvironment [11]. GBM cells can migrate along blood vessels to infiltrate the surrounding brain parenchyma [12], further contributing to BBB dysfunction, in part through the disruption of endothelial tight junctions [13].

Neurons constitute another critical component of the tumor microenvironment. At the invasive tumor margin, GBM cells can interact directly with neurons, form synapse-like structures, and integrate into neural circuits, while concomitantly upregulating genes associated with neuronal identity [14]. In contrast, more differentiated GBM cells exhibit a reduced neuronal gene expression profile [14]. Emerging evidence suggests that this functional and structural integration into neuronal networks actively contributes to tumor progression and invasiveness [15].

These findings highlight the central role of the tumor microenvironment, especially the interactions with endothelial and neuronal cells, for GBM homeostasis and progression. Thus, investigating GBM cells within a complex and heterogeneous microenvironment is essential to uncover relevant molecular mechanisms governing their behavior and to identify novel therapeutic strategies targeting tumor cell invasion.

In this study, we developed a human cerebral organoid model from induced pluripotent stem cells (hiPSCs) to investigate GBM cell interactions, infiltration, and tissue tropism. By combining cortical (COs) and endothelial organoids (EOs) into cortico-endothelial assembloids (CO+EOs), we generated a complex neuronal tissue incorporating vessel-like structures that recapitulate some features of the BBB. Co-culture with patient-derived GBM cells provides a physiologically relevant system to investigate tumor invasion, migration, morphology, molecular signatures, and interactions with neural and vascular components. We recently showed that the RNA-binding protein CELF2, a member of the CELF/Bruno-like protein family [16], is essential for maintaining expression of GSC markers (NANOG, SOX2, OLIG2), stemness, and tumorigenic potential [10]. In GBM samples, CELF2-positive tumor cells are predominantly located in highly proliferative tumor core regions, whereas CELF2-negative cells are enriched in non-dividing areas [10].

To investigate the role of CELF2 in GBM cell infiltration and tropism, we used our CO + EO assembloid model. Our results reveal that CELF2-expressing and -depleted GBM cells show distinct tropism toward neuronal and endothelial compartments, respectively. CELF2-expressing cells preferentially target neuronal regions, where they induce neuronal apoptosis and alter BBB-like tight junction structure. In contrast, loss of CELF2 promotes a transition to a non-tumorigenic, mesenchymal-like state [17], which shows minimal impact on either neuronal or EO components. Furthermore, we identify AHNAK [18] as a key effector of vascular tropism in CELF2-deficient cells, establishing the CO + EO model as a valuable platform to dissect GBM progression and tumor–microenvironment interactions.

## Materials and methods

See supplemental material and methods for detailed procedure.

### hiPSC culture

HMGU1 hiPSCs, derived from neonatal foreskin fibroblasts (ATCC CRL-2522, BJ), were kindly provided by Dr. Drukker (HMGU, Germany) under a Material Transfer Agreement (MTA), and confirmed mycoplasma-free. Cells were maintained on Matrigel-coated plates (Corning, 354234) in mTeSR1 medium (STEMCELL Technologies, #85850) with daily medium changes. Passaging was performed using Versene (Thermo Fisher, 15040066) or, for single-cell dissociation, Accutase (Sigma, A6964). For single-cell culture, 10 µM ROCK inhibitor Y-27632 was added for 24 h post-dissociation to prevent apoptosis.

### GBM cell line culture

GB5 cells were isolated from primary GBM surgical specimens (University Hospital of Nice), and TG6 cells were kindly provided by Dr. Hervé Chneiweiss (Université Pierre et Marie Curie, Paris). Cells were cultured as previously described [10]. CELF2 knockdown (shCELF2) and control (shCTRL) lines were generated as previously described [10].

### hiPSC and GBM cell line quality controls

HMGU1 and GSCs were tested annually for mycoplasma contamination by PCR using Myco_F1 and Myco_R1 primers on supernatant collected after 48 hours of culture. Genetic and chromosomal integrity of HMGU1 was evaluated with the hPSC Genetic Analysis Kit (Stem Cell Technologies, #07550), using WT-T12 and PGP1 hiPSC lines as controls. Genomic DNA was extracted from Proteinase K-lysed cell pellets, followed by isopropanol precipitation and ethanol washing. Due to recurrent mutations and chromosomal abnormalities after extended culture, HMGU1 cells were not used beyond passage 26. Pluripotency was confirmed via OCT4 immunostaining prior to downstream applications.

### Neural differentiation of cortical organoids (COs)

HMGU1 cells (25–50k/well) were seeded on Matrigel-coated 24-well plates in mTeSR1/+ until ~90% confluency, then switched to neural progenitor patterning medium (NPPM) with BMP, TGFβ, and WNT inhibitors (LDN-193189, SB-431542, XAV-939).

At days 7–8, cells were dissociated (Accutase), seeded into 96-well U-bottom plates (60k/well) in NPPM + ROCK inhibitor, and centrifuged to form aggregates. By days 8–9, aggregates were embedded in Matrigel droplets and cultured in bioreactors with NPPM + XAV-939 (LDN and SB removed from days 9 to 10).

At day 20, medium was switched to Neural Differentiation Medium (NDM), then to Long-Term Survival Medium (LTSM) by days 30–35. Medium was refreshed every 3–4 days. From days 50–60, large organoids were sectioned monthly to prevent necrosis.

### Mesoderm differentiation for EO production

Mesodermal differentiation was adapted from Wimmer et al. [19]. Dissociated hiPSCs were seeded into ultra-low attachment 96-well plates (4000 cells/well) in mTeSR1 medium with 5 µM Y-27632 (Tocris) and centrifuged at 125×*g* for 4 min to promote embryoid body (EB) formation. After 2 days, the medium was replaced with DMEM/F12 supplemented with N2/B27 (with vitamin A), 10 µM CHIR99021 (Tocris), and 30 ng/mL BMP4 (ThermoFisher) for mesoderm induction. On day 5, EBs were transferred to N2/B27 medium containing 40 ng/mL VEGF-A and 2 µM forskolin (Tocris) for 2 additional days. For vascular organoid generation, 10–15 mesodermal EBs were embedded in Collagen I/Matrigel (Corning) matrices in 12- or 24-well plates. StemPro34 SFM (ThermoFisher) with 10% FBS, 40 ng/mL VEGF-A, and 20 ng/mL FGF-2 was added to induce vessel sprouting, typically observed within 2–3 days. Medium was partially changed every other day. EOs were harvested after day 10 using modified pipette tips and gentle mechanical dissociation, and used for assembloid formation upon confirmation of vascular sprouting.

### Cortico-endothelial assembloid (CO+EOs) formation

Cortical (CO) and EO were co-cultured in low-adhesion 96-well plates in LTSM + VEGF-A (40 ng/mL) and FGF2 (20 ng/mL). Medium was partially refreshed daily. After 1 week, 10,000 GBM cells were added per well, followed by brief centrifugation (300 rpm, 1 min) and 24 h incubation. Assembloids were then transferred to an orbital shaker for continued culture.

### Immunostaining on cryostat sections

Organoids and assembloids were fixed in 4% PFA (4 °C, 4 h), cryoprotected in 1–25% sucrose, embedded in OCT, and cryosectioned (12 µm). Sections were dried, washed with PBS, and antigen retrieval was performed (0.1 M sodium citrate, 95 °C, 10 min). Sections were blocked (PBS + 5% serum + 0.3% Triton X-100) and stained using primary antibodies (≥ 4 h RT or overnight at 4 °C), Alexa Fluor secondaries (1:500), and Hoechst (1:1000). Imaging was performed with a Vectra Polaris, Zeiss Apotome, or LSM710 confocal microscopes, with analysis in HALO, Fiji, or Photoshop.

### Immunostaining image analysis on HALO software

Tissue segmentation was performed using the Random Forest Classifier in HALO. The AI was trained on marker expression to identify cortex (MAP2/TUJ1), tumor (GFP), and endothelium (CD31 + /MAP2–). Once trained, the classifier was applied to additional sections. HighPlex FL modules (v4.2.3/4.2.14) were used for marker-positive cell detection and further analysis.

### Immunostaining and tissue clearing of whole organoids

Fixed organoids were permeabilized (0.5% CHAPS, 3 h, 37 °C), blocked overnight (PBS-azide + 0.3% Triton X-100 + 5% NBCS), and incubated with primary (1:250, 2–3 days) and secondary (1:500, 24–48 h) antibodies. After washing, organoids were cleared in AKS solution (DMSO, TDE, sorbitol, Tris base) for ≥24 h, and embedded in 0.8–0.9% agarose in AKS for 3D imaging via Zeiss Lightsheet 7. Analysis was done in Imaris (v9.6).

### Immunostaining of cultured cells

Cells on coverslips were labeled with primary antibodies (see Supplementary Table 1), followed by Alexa Fluor-conjugated secondary antibodies (1:1000). Controls without primary or with non-specific antibodies were included. Imaging was done using a Zeiss AxioObserver microscope or Vectra Polaris scanner.

### Western blot

Proteins were extracted from cells/organoids, separated by SDS-PAGE, and transferred to PVDF membranes. Detection was performed with HRP-conjugated secondary antibodies and ECL reagents. Signals were imaged (Fusion FX7) and quantified using FIJI.

### RT-qPCR

RNA was extracted (TRI-Reagent), reverse-transcribed (MMLV), and analyzed by SYBR Green-based real-time PCR. Gene expression was normalized to 36B4 using the ΔΔCt method. Primer sequences are listed in Supplementary Table 2.

### Statistical analysis

Data were analyzed using Excel, GraphPad Prism, or BiostaTGV, with results expressed as mean ± SEM. Cell counts were done manually or via AI (HALO). Statistical tests included Mann–Whitney, *t* tests, and ANOVA (*P* < 0.05).

### AHNAK knockdown

GBM cells were transfected with shRNA Clone sets of three constructs against Human AHNAK nucleoprotein in psi-LVRH1H, including a scrambled control clone (ref # CS-HSH111706-LVRH, Labomics S.A., Nivelles, Belgium) using the jet Prime transfection kit (Polyplus-transfection S.A., Illkirch, France) following the recommendations of the manufacturer. Transfected cells were selected and further grown with hygromycin (50 µg/ml). The absence of AHNAK expression was analyzed by western blots as previously described.

### RNA-seq analysis

Bulk RNA-seq from shCTRL and shCELF2 GB5 cells [10] was analyzed in R (ggplot2); differential gene expression and volcano plots were generated using Neftel et al. [17] and Verhaak et al. [19] signatures.

### Histological analysis of paraffin-embedded patient-derived GBM tissues

Paraffin-embedded patient-derived GBM samples were sectioned at 4-μm thickness and processed for hematoxylin and eosin (H&E) staining as well as immunostaining using MIB1 and GFAP antibodies. Necrotic areas were identified and annotated according to established histopathological and morphological criteria, including loss of tissue architecture with disappearance of normal cellular organization, reduced cellular density with loss of viable nuclei, and the presence of cellular debris. Blood vessels were identified based on characteristic vascular morphological features, including the presence of endothelial-lined luminal structures, recognizable vessel walls, and abnormal vascular architecture typically associated with GBM, such as dilated or irregular vascular profiles and microvascular proliferation.

## Results

### hiPSC-derived CO+EO assembloids as an in vitro model of highly vascularized brain tissue

**Fig. 1:**
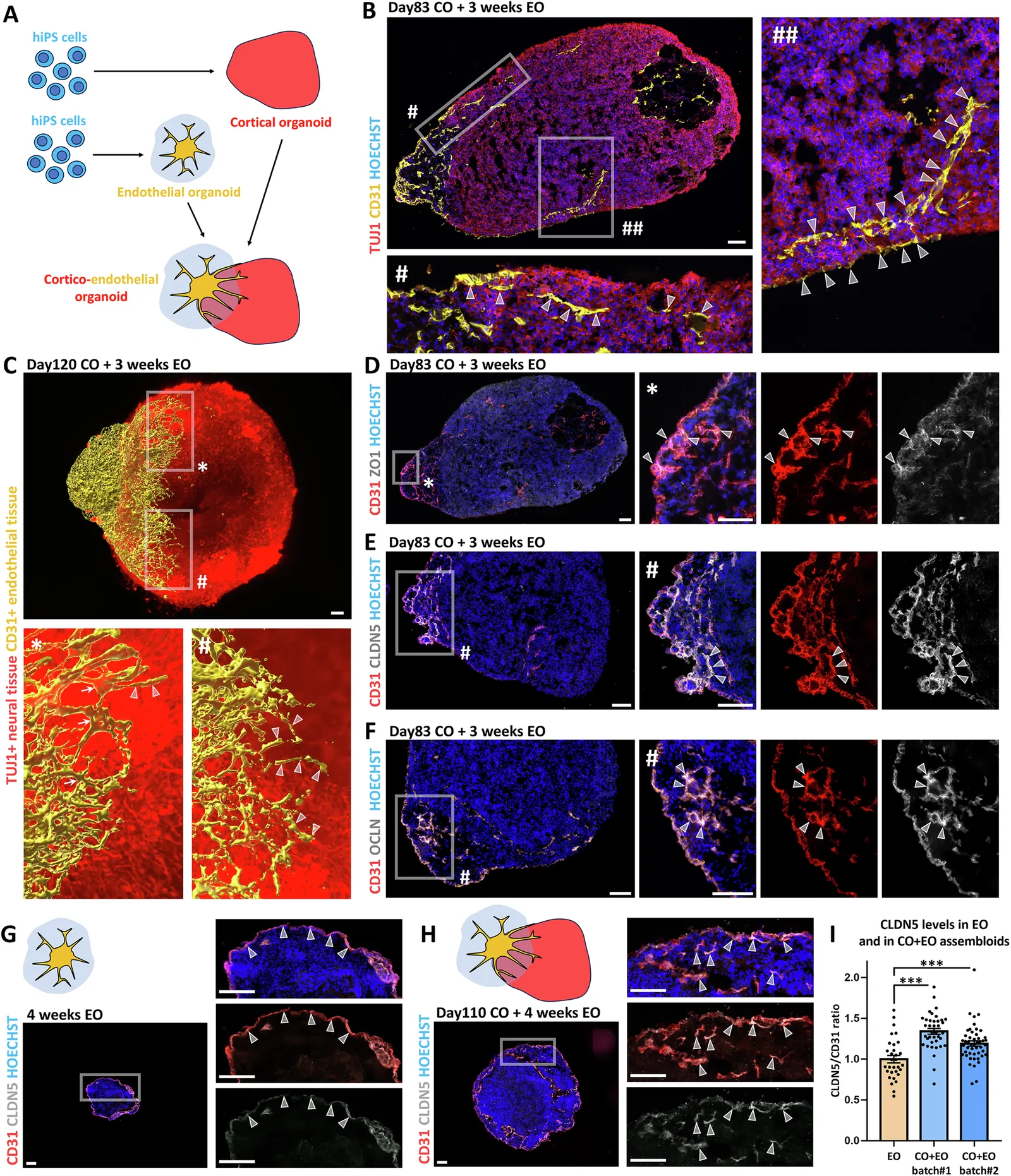
Cortico-endothelial assembloids develop blood vessel-like structures expressing BBB markers. **A** Schematic representation of the strategy used to fuse hiPSC-derived cortical organoids (COs; red) with hiPSC-derived endothelial organoids (EOs; yellow), generating cortico-endothelial assembloids (CO+EOs). **B** MAP2 (red; neural tissue) and CD31 (yellow; endothelial cells) immunostaining of a CO + EO 3 weeks after fusion. Arrowheads point to CD31+ cells forming elongated tubular structures within the neural tissue. **C** 3D reconstruction of the endothelial network (yellow) developing into neural tissue (red) in a CO + EO assembloid after tissue clearing and CD31/TUJ1 immunostaining. Arrowheads point to sprouting endothelial cells, while white arrows indicate branching points in complex ramifications. **D**–**F** Co-expression of tight junction markers ZO-1 (white in (**D**)), CLDN5 (white in (**E**)) and OCLN (white in (**F**)) with the endothelial cell marker CD31 (red); arrowheads highlight double-labeled cells in CO+EOs. **G**–**I** Double immunostaining for CD31 (red) and CLDN5 (white) in EOs (**G**) and CO + EO assembloids (**H**); white arrowheads in high-magnification images point to double-labeled blood vessel-like structures. Graph in (**I**) shows quantification of CLDN5 levels in endothelial cells, measured as a ratio of CLDN5 pixel intensity over CD31 signal, from one EO batch and two independent CO + EO batches. *P* value = 4.12 × 10^−7^ between EO and batch 1; *P* value = 0.0002878 between EO and batch 2. Scale bars: 100 µm.

To model the human brain–vascular interface, we generated cortico-endothelial assembloids (CO+EOs) by fusing 3D cortical organoids (COs) and EOs derived from the same hiPSC line (Fig. 1A). COs were produced using our telencephalic induction protocol [20]. To generate EOs, we adapted a previously described protocol [21] (Supplementary Fig. 1). Early mesodermal induction in EOs was confirmed by transient expression of the mesodermal marker Brachyury (BRY). Within 2 weeks, EOs formed capillary-like networks composed of elongated cells expressing endothelial markers (CD31, VE-CADHERIN) and VEGF receptors, suggestive of blood vessel-like structures (Supplementary Fig. 1A–C). VEGF receptor expression (VEGFR1, VEGFR2, NRP1) increased during differentiation and peaked at maturation, consistent with endothelial commitment. Immunostaining confirmed robust endothelial maturation (CD31 + , VE-CADHERIN + ) and absence of neural contamination (MAP2) (Supplementary Fig. 1D–F), indicating lineage specificity. Furthermore, PDGFRβ detection by western blot and immunostaining confirmed abundant pericyte production in EOs (Supplementary Fig. 1G, H), indicating the generation of heterogeneous, BBB-like cell populations.

To generate cortico-endothelial assembloids (CO+EOs), we fused cortical organoids (COs) aged 2 months or older (typically up to 4 months) with 2-week-old EOs (Fig.1A). Within 1 week of co-culture, endothelial cells began migrating along the CO surface (not shown), and by 3 weeks, they had efficiently infiltrated the organoid parenchyma (Fig. 1C), forming a dense, branched CD31+ network resembling blood vessel-like structures (Fig. 1D). 3D reconstruction of tissue-cleared assembloids revealed extensive integration of vascular branched structures throughout the cortical zone (Fig. 1D), while orthogonal plane analysis by light-sheet microscopy showed that CD31+ endothelial cells formed lumen-like structures reminiscent of blood vessels (Supplementary Fig. 1I). Interestingly, in addition to endothelial cells and pericytes, CO+EOs also contain GFAP+ astrocytes located in close proximity to CD31+ endothelial cells (Supplementary Fig. 1J). To assess BBB-like features, we examined tight junction markers in the endothelial compartment. CO+EOs exhibited strong expression of BBB-associated proteins Zonula Occludens-1 (ZO-1), Claudin-5 (CLDN5), and Occludin (OCLN) (Fig. 1E–G). Notably, CLDN5 levels were significantly higher in CO+EOs than in EOs alone (Fig. 1H–J), indicating that neural–endothelial interactions promote tight junction formation and BBB-like features. Although this model does not fully recapitulate a functional perfused BBB, these findings demonstrate that CO + EO assembloids spontaneously develop vascularized neuronal tissue displaying BBB-like features, including endothelial cells, astrocytes, pericytes, and neurons.

Together, these results establish CO + EO assembloids as a robust in vitro human model to study brain–vascular interactions and key aspects of BBB formation.

### CELF2-dependent tropism of GBM cells in CO+EO assembloids

**Fig. 2:**
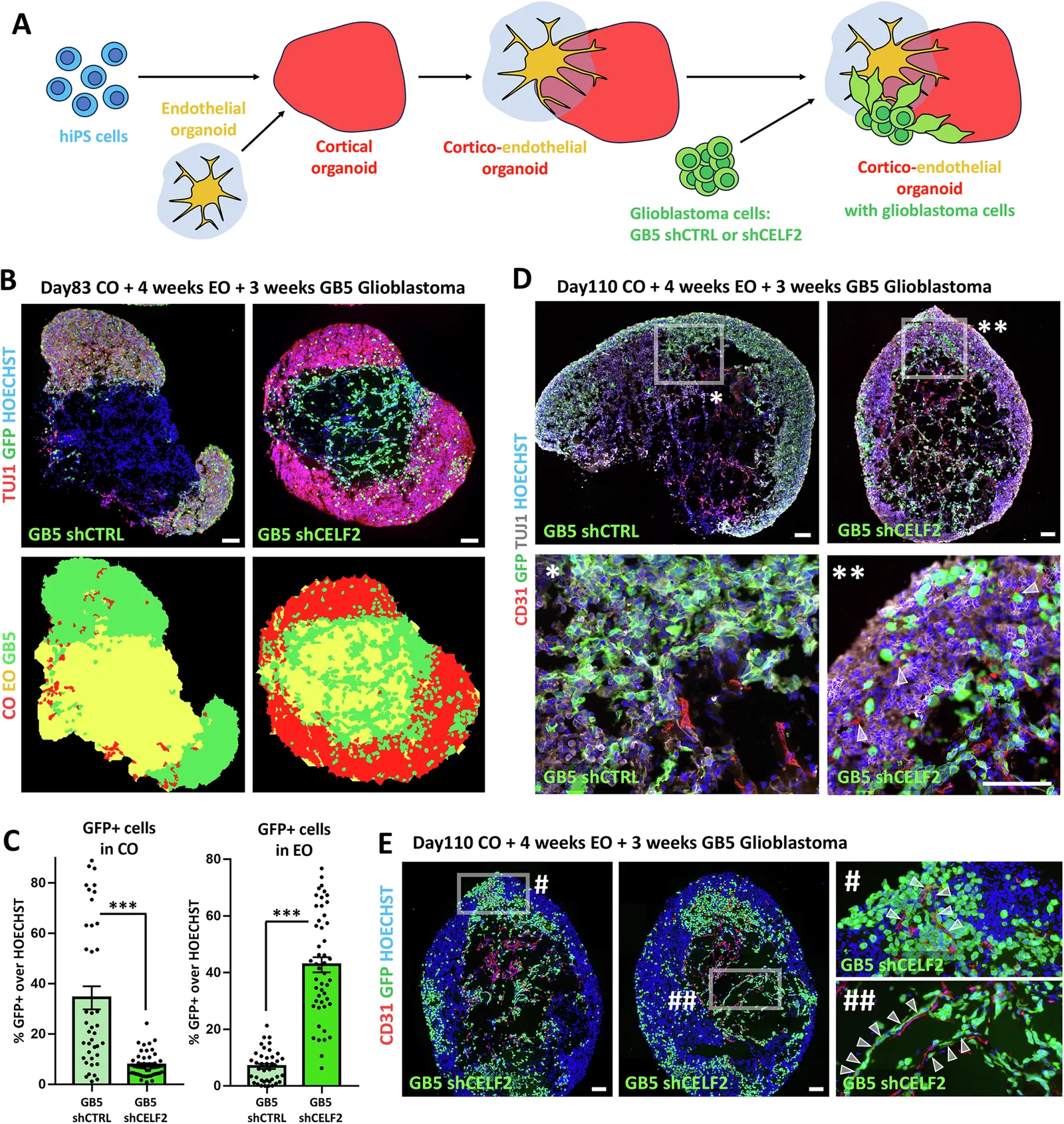
CELF2-dependent tropism of GBM cells in cortico-endothelial human assembloids (CO + EOs). **A** Schematic representation of the co-culture strategy generating cortico-endothelial assembloids (CO+EOs) with GB5 shCTRL and shCELF2 cells. **B**, **C** TUJ1 (red; neural tissue) and GFP (green; GB5 cells) immunostaining of CO+EOs, 4 weeks after fusion between EOs and COs and 3 weeks post-addition of GBM cells. Automated recognition of tumor (green), cortical (red), or endothelial (yellow) surfaces is shown in (**B**), lower row. **C** Displays the percentages of GFP + GBM cells found in each surface type in human assembloids 3 weeks after GBM invasion, with control GB5 shCTRL cells colonizing the cortical domain and CELF2-deprived shCELF2 GB5 cells preferring the endothelial part. *P* value = 3.17 × 10^−7^ between shCTRL and shCELF2 in CO; *P* value = 8.44 × 10^−11^ between shCTRL and shCELF2 in EO. **D** Triple immunostaining for CD31 (red), GFP (green), and TUJ1 (white) of CO+EOs after 3 weeks of co-culture with GB5 shCTRL or shCELF2 cells, as indicated; arrowheads point to CELF2-deprived GFP+ cells in proximity to CD31+ blood vessel-like structures. **E** CD31 (red; endothelial cells) and GFP (green; tumor cells) immunostaining of CO+EOs after 3 weeks of co-culture with GB5 shCTRL or shCELF2 cells, as indicated. Arrowheads indicate shCELF2 GB5 cells entering the cortical tissue by migrating along CD31+ endothelial cells (#) or elongating along them (##). Scale bars: 100 µm.

Given the established role of CELF2 in regulating GSC aggressiveness [10], we investigated its influence on GSC infiltration within a complex tissue environment. To this end, we co-cultured previously characterized patient-derived CELF2-expressing GSCs (GB5 shCTRL) and CELF2-depleted counterparts (GB5 shCELF2) with cortico-endothelial assembloids (CO+EOs) (Fig. 2A). CELF2 knockdown was confirmed both in cultured GBM cells (Supplementary Fig. 2A) and by immunostaining of CO + EO sections after 3 weeks of co-culture (Supplementary Fig. 2B). First, we segmented the immunostained surface of GBM-invaded assembloids into neural and endothelial regions and quantified tumor cell distribution (Fig. 2B, C). CELF2-expressing cells preferentially localized to TUJ1+ neuronal zones, while CELF2-deficient cells accumulated in endothelial regions (Fig. 2B, C and Supplementary Fig. 3A, B). This spatial divergence correlated with a loss of tumor-free MAP2 + /TUJ1+ areas in organoids invaded by shCTRL cells and a reduction in tumor-free endothelial zones in organoids invaded by shCELF2 cells (Supplementary Fig. 3B).

Interestingly, CELF2-deficient GSCs often aligned with CD31+ structures, even when present in neural regions, suggesting that endothelial cells may guide their invasion (Fig. 2D, E). These cells also displayed an elongated, migratory-like morphology when interacting with vasculature (Fig. 2E). We repeated these experiments using a second patient-derived line, TG6, with or without CELF2 knockdown. Consistent with the GB5 findings, TG6 shCTRL cells favored neural zones, whereas TG6 shCELF2 cells were redirected to endothelial compartments (Supplementary Fig. 3C, D). Notably, Ki67 staining indicated that tumor cells remained non-quiescent within the organoid, regardless of CELF2 status. However, CELF2 repression was associated with a marked reduction in OLIG2⁺ mitotic cells (Supplementary Fig. 3E, F), consistent with a shift toward an OLIG2-negative cellular state. This observation is particularly relevant in the context of our study, as OLIG2 is a well-established marker of GBM stemness and aggressiveness, and we previously demonstrated that its expression depends on CELF2 activity in GSCs [10].

Overall, these results reveal a CELF2-dependent tissue tropism: CELF2-expressing GSCs target neural tissue, while CELF2-deficient cells are redirected toward the vasculature. These findings further highlight the CO + EO assembloid as a powerful model for dissecting molecular mechanisms underlying GBM infiltration and tissue tropism.

### Human COs reveal CELF2-dependent dynamics of GBM invasiveness in neural tissue

**Fig. 3:**
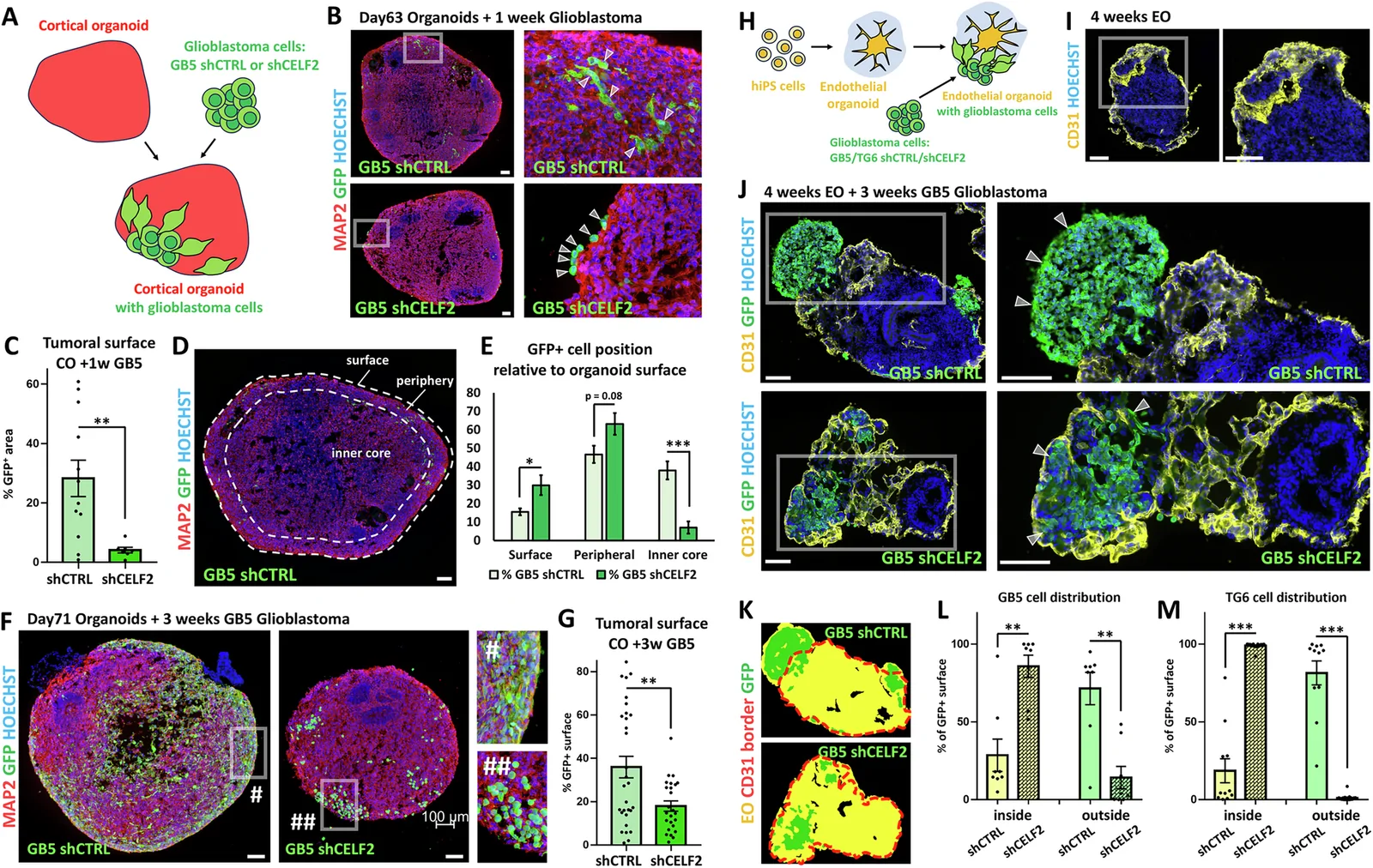
GBM cell invasive potential in cortical or endothelial organoids. **A** Schematic representation of cortico-glioblastoma (GBM) co-culture integrating GB5 control (shCTRL) or CELF2-deprived (shCELF2) GB5 cells into COs. **B**, **C** MAP2 (red) and GFP (green) immunostaining of day 63 COs supplemented with shCTRL and shCELF2 GB5 cells after 1 week of co-culture. White arrowheads in high-magnification insets point to GFP + GB5 cells; note that shCELF2 cells exhibit a round morphology and are primarily distributed on the external surface of the COs. Graph in (**C**) shows quantification of the GFP+ surface area in organoid sections after 1 week of co-culture (HALO) from 12 sections (shCRTL) or 7 sections (shCELF2) from two independent organoid batches. *P* value = 0.008. **D**, **E** Evaluation of GBM cell position in organoid sections, classified as on the surface, in the periphery (100 μm from the surface), or in the inner core. Graph in (**E**) shows the distribution of GBM cell positions for shCTRL and shCELF2 assembloids after 1 week of co-culture. manual quantification (FIJI/Photoshop) *n* = 8 sections from *n* = 6–8 organoids, from two independent batches. *P value = 0.0240; ***P value = 0.00029. **F**, **G** MAP2 (red) and GFP (green) immunostaining of assembloids after 3 weeks of co-culture. The graph in G shows quantification of the GFP+ surface area. automated quantification (HALO) from 25 sections from *n* = 6–8 organoids from five independent batches; *P* value = 0.003399. **H** Schematic representation of endothelial organoids (EOs; yellow) and GBM cells (green) in co-culture. **I**, **J** CD31 (yellow; endothelial cells) and GFP (green; tumoral cells) immunostaining of EOs after 4 weeks of in vitro differentiation (**I**) and 3 weeks of co-culture with GBM cells (**J**). **J** Shows exemplificative organoid sections of EOs co-cultured with GB5 shCTRL and shCELF2 cells. Note that shCELF2 GB5 cells exhibit tropism toward endothelial tissues and demonstrate high invasive potential into CD31+ domains, whereas shCTRL cells tend to form peripheral aggregates and invade less. Arrowheads indicate clusters of GFP+ cells. **K**–**M** HALO software automated recognition of endothelial domains (yellow) and GFP + GBM cells (green). Graphs in (**L**, **M**) show the percentages of GB5 (**L**) and TG6 (**M**) GFP+ cells found inside or outside the borders of endothelial areas (red lines in (**K**)). **L** For both shCTRL and shCELF2, automated quantification HALO from *n* = 8–9 sections from *n* = 3–5 organoids from two independent batches. shCTRL vs shCELF2 in EO; *P* value = 0.0039; shCTRL outside vs shCELF2 in EO; *P* value = 0.0039. Scale bars: 100 µm.

To specifically assess the influence of CELF2-dependent invasive potential of GBM cells in a neural-specific context, while avoiding confounding factors introduced by the presence of endothelial tissue, we co-cultured GB5 cells with COs alone (Fig. 3A). One week after co-culture, control CELF2-expressing GSCs readily infiltrated the cerebral organoid, whereas CELF2-depleted GSCs remained largely confined to the organoid surface (Fig. 3B–E and Supplementary Fig. 4A), resulting in reduced surface occupancy in COs (Fig. 3C). Morphologically, CELF2-deficient cells displayed a rounded, non-migratory phenotype within the neural environment (Supplementary Fig. 4B), consistent with their reduced invasive potential and in contrast to their elongated, migratory morphology observed during endothelial invasion. In contrast, CELF2-expressing cells retained an elongated morphology, characteristic of actively migrating cells (Fig. 3B). To directly assess GSC infiltration dynamics, we quantified the spatial distribution of tumor cells across the organoid depth. By 2 weeks, CELF2-expressing cells had migrated into the organoid core, while CELF2-depleted GCSs remained largely confined to the surface or peripheral regions (Fig. 3D, E). These differences became even more pronounced at 3 weeks, with CELF2-expressing GB5 cells forming extensive, proliferative aggregates deep within the cortical tissue, whereas CELF2-deficient cells formed smaller, restricted clusters at the periphery (Fig. 3F), resulting in reduced tumor area occupancy (Fig. 3G). Light-sheet microscopy of tissue-cleared COs with automated detection of GFP+ cells confirmed that CELF2-expressing tumor cells penetrated the organoid core, whereas CELF2-depleted cells remained predominantly localized at the outer edge (Supplementary Fig. 4C). Consistent with findings obtained in CO + EO assembloids (Supplementary Fig. 3E, F), the CELF2-depleted cell population co-cultured with COs alone was not quiescent but displayed reduced infiltration into the neural tissue. This was accompanied by a marked decrease in OLIG2⁺ mitotic cells (Supplementary Fig. 4D, E), consistent with a shift away from an OLIG2-driven progenitor-like state rather than a global impairment of proliferative capacity. Similar results in terms of changes in GBM invasive potential upon CELF2 modulation were obtained using the TG6 GBM cell line (Supplementary Fig. 5A–E). These findings confirm that CELF2 enhances GBM cell stemness and promotes invasive behavior within human neuronal tissue.

### CELF2-dependent GBM tropism toward human endothelial tissue

To further validate the endothelial tropism of GBM cells driven by CELF2 depletion, initially observed in CO + EO assembloids, we investigated GBM behavior in an endothelial-only context by co-culturing CELF2-expressing (shCTRL) and CELF2-depleted (shCELF2) GB5 or TG6 cells with EOs alone (Fig. 3H). The presence of mature endothelial cells within the EOs was confirmed by CD31 immunostaining after 4 weeks of in vitro differentiation (Fig. 3I). GBM cells were subsequently introduced and co-cultured with the EOs for an additional 3 weeks to allow their integration and to assess their capacity for adhesion and infiltration. In this simplified setting, shCELF2 cells exhibited enhanced integration into the capillary-like vascular structure, adhering to and infiltrating the endothelial network (Fig. 3J and Supplementary Fig. 5F). In contrast, shCTRL cells largely failed to penetrate the EOs and instead formed compact aggregates along the organoid periphery (Fig. 3J and Supplementary Fig. 5F), displaying behavior distinct from that observed in COs or CO+EOs. To quantify these differences, we employed AI-based image analysis (HALO software), to define the endothelial region within the EOs and assess the spatial distribution of GBM cells (Fig. 3K). This analysis revealed a significantly higher number of CELF2-depleted cells located within the endothelial region for both GB5 and TG6 cell lines, compared to their CELF2-expressing counterparts (Fig. 3L, M).

These findings show that while CELF2 repression does not impair the migratory capacity of GBM cells, it reprograms their tropism toward endothelial compartments, highlighting a role for CELF2 in regulating tissue affinity rather than cell motility. Collectively, these findings underscore the role of CELF2 in directing GBM cell tropism toward neural rather than vascular regions, revealing distinct tissue- and CELF2-dependent patterns of invasion.

### Patient tumor analysis confirms CELF2-dependent GBM cell tropisms identified in CO+EO assembloid models

**Fig. 4:**
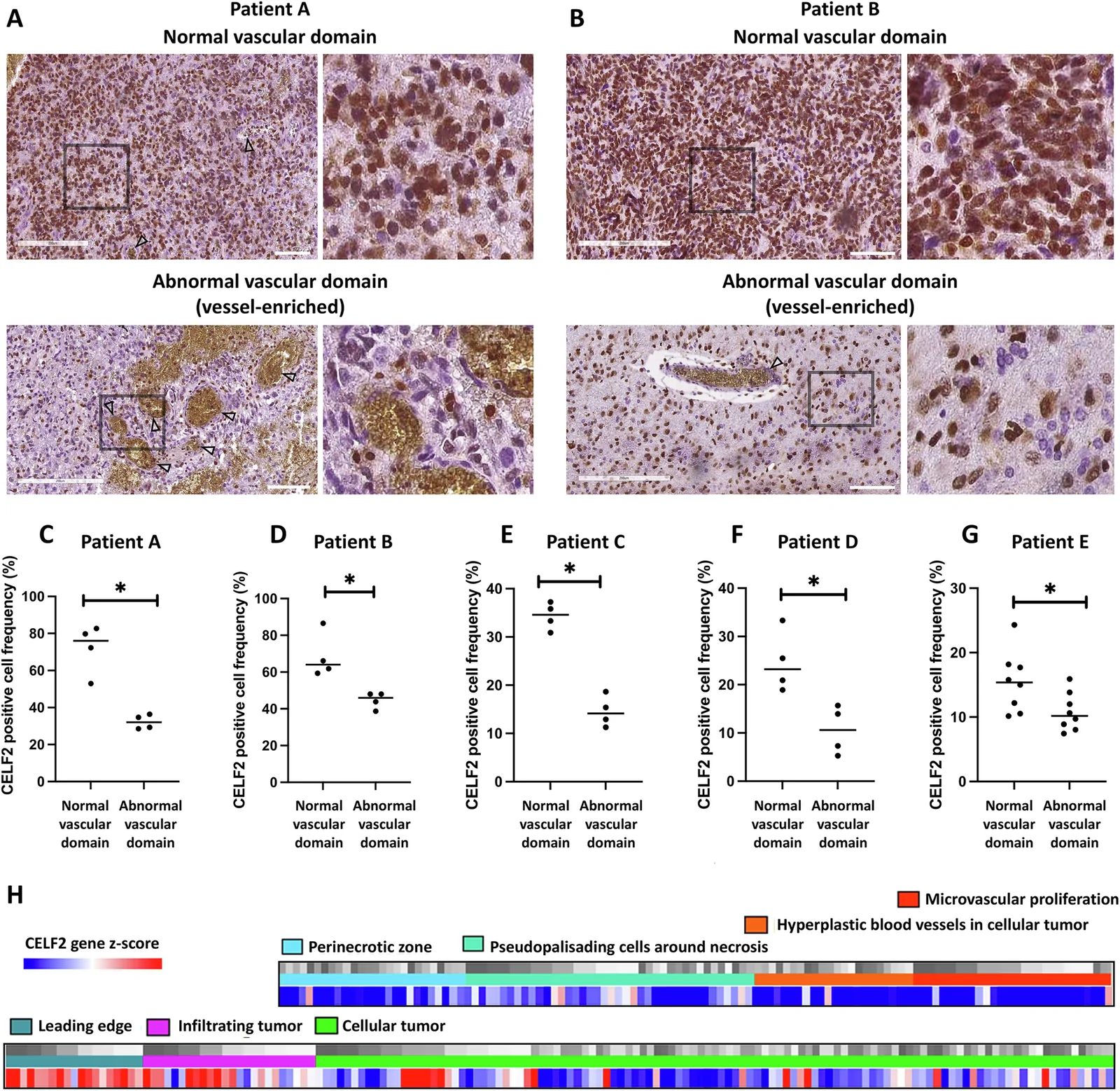
CELF2+ cell percentage in normal or abnormal vascular domains in GBM patient samples. **A**, **B** Exemplary immunohistochemistry stainings of GBM samples from two patients, showing CELF2 (brown) over hematoxylin-eosin staining that highlights nuclei (violet) in areas with few and small blood vessels (normal vascular domains; upper row) and in areas with large and abnormal blood vessels (vessel-enriched abnormal vascular domains; bottom row). Arrowheads highlight blood vessels. CELF2 immunohistochemistry stainings on the other three GBM patients are shown in Supplementary Fig. 5. **C**–**G** Graphs showing the percentages of CELF2+ cells in normal and abnormal vascular domains within the GBM tumor sections of five different patients. **H** CELF2 ISH z-scores derived from the Ivy-GAP dataset (GlioVis), showing CELF2 expression across histologically annotated GBM microenvironments. CELF2 is upregulated at the leading edge and in infiltrating tumor regions, and downregulated in endothelial-associated microenvironments, such as hyperplastic blood vessel-enriched areas. *P* value < 0.05. Scale bars: 100 µm.

We next examined the pathophysiological relevance of the CELF2-dependent tropism observed in CO + EO assembloids by analyzing human GBM tissue samples. As expected, given GBM intra-tumoral heterogeneity, non-mitotic tumor regions, often adjacent to necrotic zones, showed strong immunoreactivity for the astrocyte differentiation marker GFAP and were frequently associated with abnormal dense vasculature (Supplementary Fig. 6A, B). In contrast, mitotic tumor regions were characterized by sparse vascularization (Supplementary Fig. 6A, B). To assess the spatial distribution of CELF2-positive GBM cells, we performed immunohistochemical staining on tumor sections from five different GBM patients using a CELF2-specific antibody, and quantified the proportion of CELF2-positive cells in vessel-enriched abnormal vascular domains versus poorly vascularized regions (Fig. 4A–G). Strikingly, CELF2-positive cells were significantly enriched in poorly vascularized areas and markedly under-represented in vessel-enriched abnormal regions (Fig. 4A–G and Supplementary Fig. 6C–E). This inverse correlation between CELF2 expression and vascular density was consistent and statistically significant across all patient samples analyzed. Data from the Ivy Glioblastoma Atlas Project (Ivy-GAP), showing CELF2 expression profiles across histologically annotated GBM microenvironments, confirmed increased CELF2 expression at the leading edge and within infiltrating tumor regions, together with reduced expression in endothelial-rich microenvironments (Fig. 4H). These in vivo findings closely parallel our in vitro observations in CO + EO assembloids, where CELF2-expressing GSC preferentially localized to neural compartments rather than endothelial zones. Together, these data establish a strong correlation between the CO + EO model and patient tumor biology, underscoring the pathophysiological relevance of the CO + EO assembloid model for dissecting the molecular and spatial determinants of GBM cell tropism.

### CELF2-dependent impact on neuronal and endothelial components of CO+EO assembloids

**Fig. 5:**
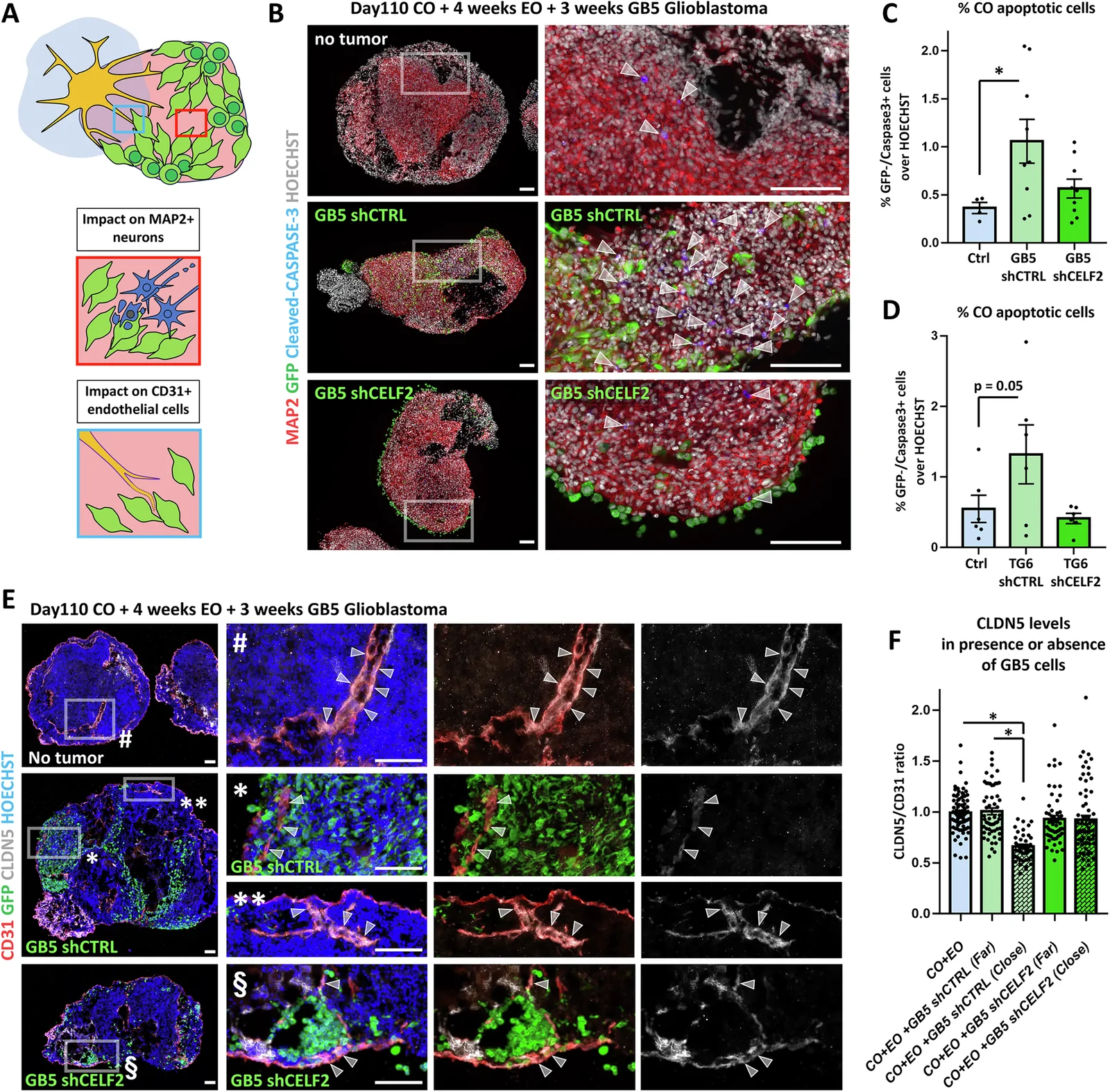
Neuronal survival and tight junction integrity in CO + EOs upon GBM cell invasion. **A** Schematic representation of a CO + EO assembloid invaded by GBM cells (green); MAP2+ neurons and CD31+ endothelial cells are the main cellular populations interacting with GBM cells evaluated here. **B**–**D** Triple immunostaining for MAP2 (red; neural tissue), GFP (green; GBM cells), and cleaved CASPASE3 (blue; apoptotic cells) in control (top row in (**B**)), GB5 shCTRL-invaded (middle row), and GB5 shCELF2-invaded (bottom row in (**B**)) CO+EOs. Graphs show the percentage of apoptotic neuronal cells (double MAP2+ and CASPASE3 + ) in control assembloids and in assembloids invaded by shCTRL or shCELF2 GB5 (**C**: *n* = 4–9 automated quantifications from *n* = 6–8 sections from three organoids per type from 1 batch; *P* value = CO + EO vs CO + EO + GB5 shCTRL *P* value = 0.0299) or TG6 (**D**: *n* = 6 automated quantifications from *n* = 6 sections from three organoids per type from 1 batch; *P* value = 0.054244094 CO + EO vs CO + EO + GB5 shCTRL) cells. **E**, **F** Triple immunostaining for GFP (green), CD31 (red), and CLDN5 (white) in CO + EO assembloids after 3 weeks of co-culture with shCTRL or shCELF2 GB5 cells; pixel intensity of CLDN5 in CD31+ cells under different conditions (shCTRL versus shCELF2 cells; far versus close to GBM cells) is displayed in (**F**), showing decreased CLDN5 levels when control CELF2-expressing cells come in close contact with endothelial cells. *P* value < 0.02. Scale bars: 100 µm.

We next investigated the impact of patient-derived GBM cell invasion on MAP2-positive neurons and endothelial cells within the CO + EO host tissue, and whether these effects depend on CELF2 expression (Fig. 5A). To this aim, we first evaluated neuronal integrity by co-staining for MAP2 and the apoptotic marker cleaved Caspase-3 (Fig. 5B). We observed increased neuronal apoptosis in CO+EOs co-cultured with CELF2-expressing GB5 or TG6 control cells compared with assembloids co-cultured with CELF2-depleted counterparts (Fig. 5B–D), indicating an impact of highly invasive GSCs on neuronal survival. We next investigated whether GBM cell infiltration could also compromise the organization of the endothelial component. To this end, we quantified the ratio of CLDN5 (a tight junction marker) to CD31 (an endothelial marker) in regions proximal or distal to GBM cells (Fig. 5E, F). No significant changes in the CLDN5/CD31 ratio were observed in regions distant from tumor cells or in any condition involving CELF2-deficient cells. However, in regions where CELF2-expressing GBM cells were in direct contact with endothelial cells, the CLDN5/CD31 ratio was significantly reduced (Fig. 5E, F), indicating a local disruption of tight junction protein CLDN5 expression. Interestingly, quantification of ZO-1 and OCCLUDIN levels in a similar experimental setting showed no significant changes following GSC invasion, suggesting a selective effect on CLDN5. Together, these results indicate that aggressive CELF2-positive GSCs exert deleterious effects on the CO + EO microenvironment by inducing neuronal apoptosis and altering CLDN5 expression in endothelial structures. In contrast, CELF2 depletion, previously shown to convert aggressive GSCs into a less invasive phenotype [10], is sufficient to prevent these deleterious effects and preserve host tissue integrity.

### Molecular signatures correlating with CELF2-dependent GSC tropism in CO+EO assembloids

**Fig. 6:**
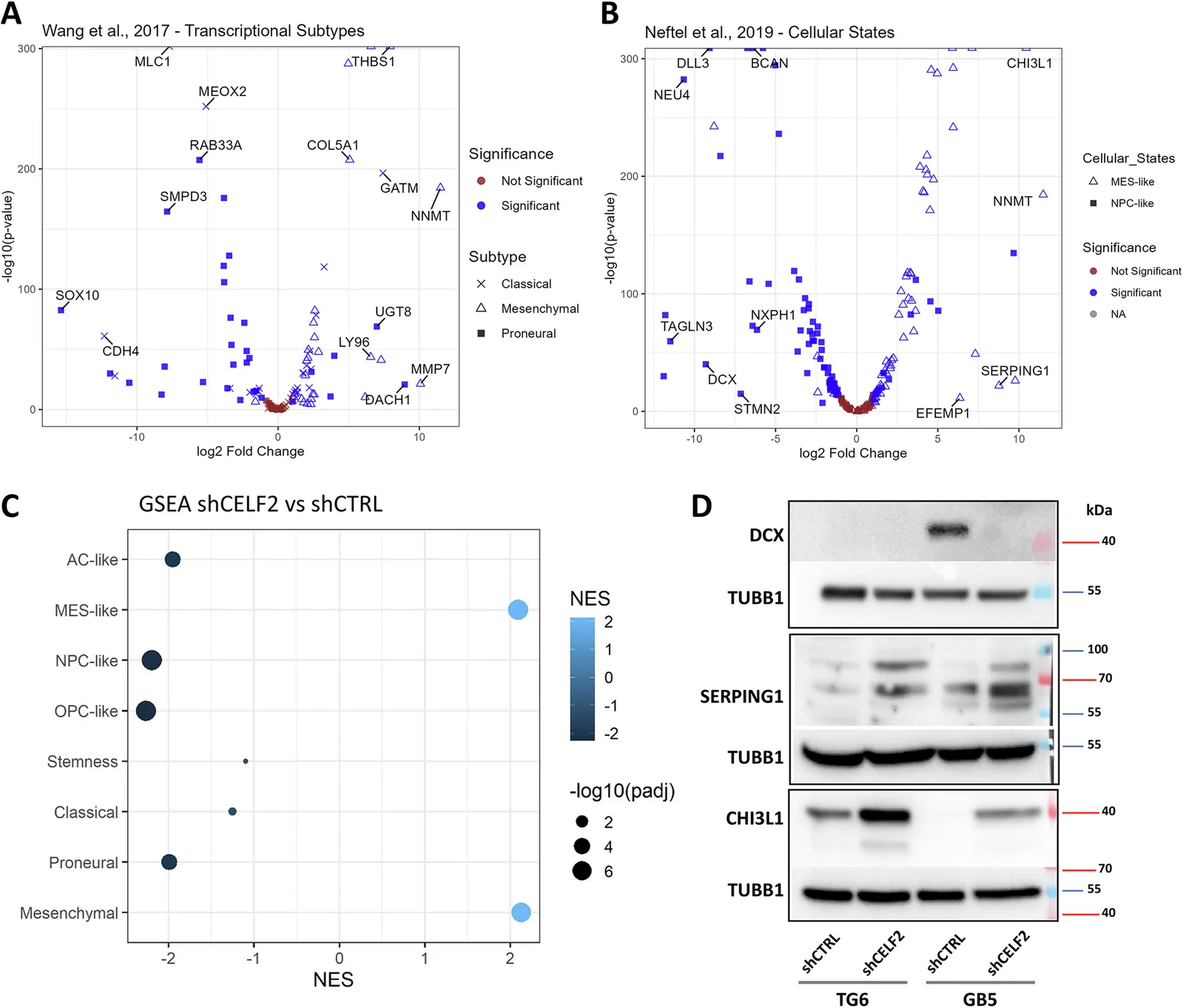
CELF2-dependent molecular signatures in GBM cells. **A**, **B** Volcano plots showing differentially expressed genes (*x* axis: fold change; *y* axis: corresponding *P* value) comparing GB5 shCELF2 and GB5 shCTRL cells; **A** is based on Verhaak/Wang transcriptomic subtype signatures (Classical, Mesenchymal, and Proneural), whereas **B** represents cellular states (MES-like and NPC-like) as defined by Neftel. **C** Gene Set Enrichment Analysis (GSEA) of cellular states, stemness, and transcriptomic subtype signatures indicates a shift toward mesenchymal-like profiles following CELF2 downregulation, with reduced expression of AC-like, OPC-like, and NPC-like states and only modest changes in stemness-associated genes. **D** Western blot analysis for the protein expression of representative genes belonging to the neural (DCX) or mesenchymal (SERPING1, CHI3L1) signatures in GB5 and TG6 cells expressing or not expressing CELF2.

To further understand how CELF2 expression influences GSCs phenotype and behavior within CO + EO assembloids at the molecular level, we re-analyzed bulk RNA sequencing data previously generated from GB5 cells with or without CELF2 expression [10]. Specifically, we examined gene expression patterns associated with classical, mesenchymal or proneural signatures as defined by Verhaak/Wang [19] (Fig. 6A), as well as neural, astrocytic, oligodendrocyte progenitor, and mesenchymal GBM subtypes, defined by Neftel et al. [17] (Fig. 6B). When analyzing Verhaak/Wang signature genes, CELF2 downregulation led to reduced expression of proneural markers accompanied by increased expression of mesenchymal markers, whereas genes associated with the classical subtype remained largely unchanged (Fig. 6A). Consistent with this observation, Neftel-based analysis revealed that CELF2-expressing GB5 cells were enriched for neuronal transcriptional programs, including genes associated with neuronal development (DCX, STMN2, TAGLN3, CRABP1, DLL3), GBM progression (NEU4, BCAN1), epilepsy-associated signaling (NXPH1), and hypoxia-related pathways (SDH) (Fig. 6A, B). In contrast, CELF2-deficient GB5 cells displayed a mesenchymal-like transcriptional profile characterized by elevated expression of CHI3L1, a key driver of mesenchymal identity [22], EFEMP1, a regulator with tumor suppressor functions [23, 24], NNMT, which promotes mesenchymal GBM proliferation [25], and SERPING1, associated with poor GBM prognosis [26] (Fig. 6B and Supplementary Table 3). Gene set enrichment analysis of both signature datasets further confirmed a shift toward a mesenchymal-like state following CELF2 downregulation (Fig. 6C). These transcriptional differences were corroborated at the protein level using representative neural and mesenchymal markers (Fig. 6D). Comparison with transcriptional datasets from Ren et al. [26] further supported the existence of two distinct CELF2-dependent transcriptional programs: one favoring a neuronal-like identity and another promoting a mesenchymal state. Genes enriched in CELF2-expressing cells were associated with synapse organization (Supplementary Table 4) and positive regulation of neural development and neurogenesis, including *NEUROD1, STMN2, SEMA5A, PCP4, NLGN3*, and *BCAN* (Supplementary Table 3). Conversely, CELF2-deficient cells upregulated genes associated with mesenchymal transformation and vascular interaction, including *CHI3L1*, *HSPG2* (a vascular extracellular matrix component), *VEGFA*, and *ADM* (both involved in vascular development and permeability) (Supplementary Table 3). Notably, several integrin genes were also upregulated in CELF2-deficient cells, potentially contributing to enhanced adhesion and endothelial tropism. Together, these findings indicate that CELF2 promotes a neural progenitor-like transcriptional state while repressing mesenchymal and vascular-associated programs, thereby shaping the distinct invasive behavior and microenvironmental preferences of GBM cells in a tissue-specific manner.

### AHNAK contributes to the endothelial tropism in CELF2-deficient GBM cells

**Fig. 7:**
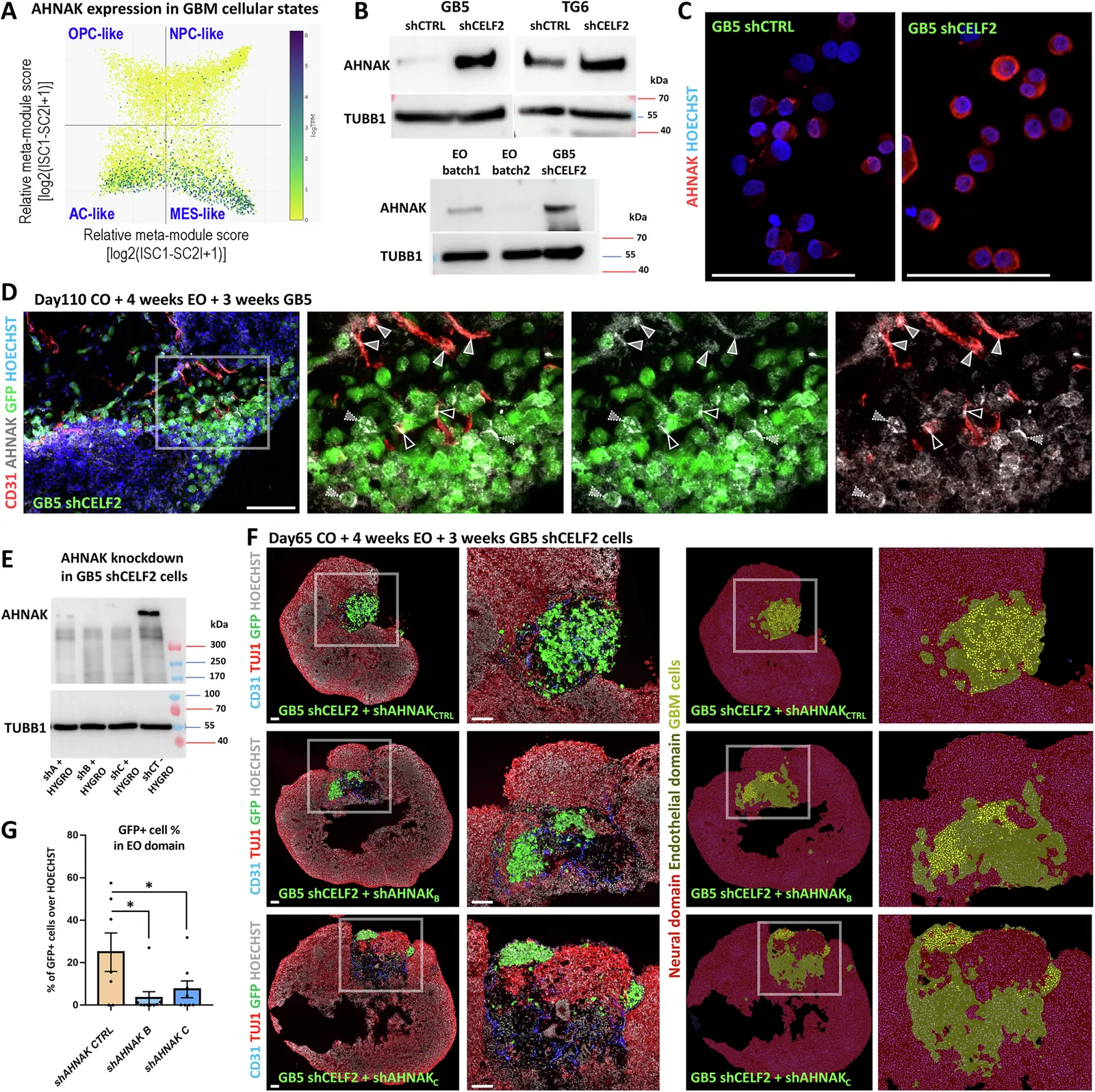
AHNAK-dependent tropism and distribution of CELF2-deprived GBM cells in CO + EOs. **A** Two-dimensional representation of AHNAK expression in distinct GBM cellular states. Each quadrant corresponds to one cellular state, and the blue dots represent the highest AHNAK expression. This representation was established using the single-cell portal of the Broad Institute (https://singlecell.broadinstitute.org). **B** Quantification of AHNAK protein levels by western blot in CELF2-depleted TG6 and GB5 cells showing marked upregulation upon CELF2 loss, along with lower but detectable endogenous expression in endothelial cells (two independent EO batches) (please refer to the original western blot file for the protein molecular weight). **C** Immunostaining for AHNAK (red) in GB5 shCTRL and shCELF2 cells, highlighting its membrane localization. **D** Confocal imaging of triple immunostaining for AHNAK (white), CD31+ endothelial cells (red), and GBM cells (green) in CO+EOs, showing AHNAK expression in both endothelial cells and GBM cells. **E** Western blot validation of AHNAK downregulation in three distinct clones of GB5 shCELF2 cells using specific shRNAs. **F**, **G** Triple immunostaining for CD31 (blue; endothelial cells), TUJ1 (red; neuronal cells), and GFP (green; GBM cells) in CO+EOs co-cultured with GB5 shCELF2 cells expressing a control scramble shRNA (shAHNAK_CTRL_) or AHNAK-targeting shRNAs (shAHNAK_B_ or shAHNAK_C_), with representative images of automated detection of endothelial domains and GFP+ cells on the right. Compared to control shCELF2 cells, AHNAK-depleted shCELF2 cells tend to cluster at the periphery of the endothelial domain instead of spreading throughout. Graph in (**G**) shows the percentage of GFP + GBM cells relative to total Hoechst+ cells within endothelial domains under the different conditions. *P* value < 0.02. Scale bars: 100 µm.

Among the genes upregulated within the mesenchymal transcriptional program of CELF2-deficient GBM cells, we focused on the large scaffold protein AHNAK to assess its potential role in mediating endothelial affinity in the CO + EO assembloid model (Fig. 7A). Western blot analyses of both GB5 and TG6 cell lines confirmed that AHNAK protein was robustly expressed in CELF2-deficient GSCs, while its expression was weak in CELF2-expressing cells and in EOs (Fig. 7B). Immunofluorescence staining revealed high levels of AHNAK in GB5 shCELF2 cells, with prominent localization at the plasma membrane (Fig. 7C). Accordingly, co-immunostaining of AHNAK and CD31 in CO + EO assembloids confirmed high AHNAK expression in shCELF2 cells, whereas endothelial cells exhibited only weak AHNAK staining (Fig. 7D and Supplementary Fig. 7), indicating that CELF2-depleted tumor cells constitute the main source of AHNAK in this model. Moreover, AHNAK-positive tumor cells were frequently observed in close proximity to CD31+ endothelial structures in assembloids (Fig. 7D), highlighting the close spatial association between CELF2-negative, AHNAK-positive tumor cells and vascular components. To determine whether AHNAK is functionally required for the endothelial tropism of CELF2-deficient GBM cells, we performed stably knockdown of AHNAK using three independent AHNAK-targeting shRNA sequences. Western blot analysis confirmed efficient AHNAK knockdown in all clones, with a near-complete depletion in clones B and C, which were selected for CO + EO co-culture experiments (Fig. 7E). CELF2-deficient cells transfected with a non-targeting shRNA were used as controls (Fig. 7E).

Following 3 weeks of co-culture, CO + EO assembloids were harvested, sectioned, and analyzed. The GFP signal from tumor cells enabled the visualization and spatial mapping of their distribution within the CO + EO assembloid (Fig. 7F). As expected, CELF2-deficient cells expressing control shRNA displayed pronounced endothelial tropism, preferentially infiltrating CD31⁺ vascular regions (Fig. 7F, upper line). In contrast, AHNAK-depleted cells formed peripheral aggregates and failed to efficiently infiltrate or distribute within the endothelial compartment, resulting in markedly reduced tumor occupancy in these regions (Fig. 7F, G). These findings demonstrate that AHNAK is a critical effector of endothelial tropism in CELF2-deficient GSCs. Moreover, they reveal that silencing AHNAK is sufficient to disrupt vascular affinity and partially reprogram GBM cell tropism within the CO + EO environment. More broadly, these results underscore the relevance of AHNAK as a functional mediator of GBM cell–vascular interactions and highlight the utility of CO + EO assembloids as a platform for dissecting the molecular mechanisms governing GBM tropism within complex cortico-endothelial environments.

## Discussion

### Modeling the GBM microenvironment using a human cortico-endothelial assembloid system

GBM aggressiveness, including therapeutic resistance, is driven by intrinsic tumor cell plasticity and by dynamic interactions with multiple cell types within the tumor microenvironment. Neurons and endothelial cells critically influence tumor cell behavior and contribute to disease progression by interacting with GBM cells and secreting factors that maintain a GSC state [27, 28]. GBM is also characterized by a highly developed but often dysfunctional vasculature, which contributes to hypoxic and necrotic tumor cores, further supporting GSC maintenance and tumor aggressiveness [29]. Several 3D in vitro models have been developed to reproduce human GBM architecture [30]. Patient-derived GBM organoids retain key histopathological features of the original tumors, including nuclear atypia, high mitotic activity, and pleomorphic nuclei [31], as well as glial and GSC markers such as SOX2 and OLIG2. However, these models generally lack healthy brain cell types, particularly neurons, and only sporadically contain CD31-positive endothelial cells, limiting the study of tumor–host interactions at the neurovascular interface, where critical mechanisms of invasion and microenvironmental remodeling occur.

By adapting and extending previously established protocols [20, 21, 32], we developed a novel human iPSC-derived cortico-endothelial assembloid model (CO + EO) that combines cortical and endothelial tissues in a 3D context. Although this model does not fully recapitulate brain cellular diversity, it provides key advantages. First, it offers a human-specific environment for patient-derived GBM cells, avoiding limitations associated with xenograft systems. Second, it incorporates mature neurons and vascular-like structures, two critical components of GBM physiopathology. Unlike HUVEC-based models, CO+EOs more closely reflect BBB-like properties, including tight junction formation [33]. This likely results from the co-development and progressive maturation of cortical and endothelial tissues over several weeks in the CO + EO system. This extended co-culture period supports the emergence of brain-like cytoarchitecture, including neuronal layers interspersed with endothelial networks expressing BBB markers such as CLDN5, OCLN, and ZO-1. These features are consistent with recent studies showing that organoid-based assembloids can recapitulate fundamental aspects of human BBB structure and function [34]. More complex BBB models incorporating HBMECs, astrocytes, and pericytes have been developed [35], yet their integration into stable 3D organoid systems remains technically challenging [36]. In contrast, CO+EOs inherently include astrocytes and pericytes within the culture system, arising through intrinsic differentiation and spontaneous co-development, which may better reflect a physiological organization.

The CO + EO model thus offers a physiologically relevant platform to explore neurovascular-like interactions and enables detailed dissection of the mechanism by which GBM cells infiltrate and remodel brain tissue at the tumor-brain interface, a critical but still poorly understood step in disease progression.

### CELF2 modulates GBM cell identity, tissue tropism, and interaction with the microenvironment

GBM comprises a heterogeneous population of tumor cells endowed with varying tumorigenic potential [37–39]. In this study, we focused on GSC populations differing in their expression of the RNA-binding protein CELF2 to determine its role in modulating malignant cell behaviors. Our CO + EO model revealed that CELF2 governs GBM cell tropism, with CELF2-positive cells preferentially infiltrating neural tissue, whereas CELF2-deficient cells show enhanced tropism toward endothelial structures. These findings are consistent with histological observations in patient samples, where CELF2-expressing GSCs are enriched in mitotically active and poorly vascularized tumor zones [10]. The elongated morphology adopted by CELF2-deficient GSCs when aligned with CD31⁺ vascular structures is particularly striking, as it includes not only an elongated cell body but also elongated nuclei, a hallmark feature of endothelial cells. This phenotypic resemblance is especially evident when tumor cells are closely associated with vasculature, even within otherwise neural regions. Such morphological convergence raises the possibility that a subset of GBM cells may undergo endothelial-like mimicry upon interaction with blood vessels. Vascular mimicry and endothelial transdifferentiation have been described in GBM [40, 41] and are thought to facilitate invasion, perivascular migration, and adaptation to the vascular niche. Although direct endothelial differentiation is not demonstrated here, the close spatial association with CD31⁺ structures and the acquisition of endothelial-like morphology suggest that CELF2-deficient GSCs may adopt structural features that facilitate vessel-guided invasion. Transcriptomic profiling further revealed that CELF2-expressing GBM cells exhibit a neuronal/synaptic gene signature [17], consistent with profiles of invasive GBM cells integrating into neural circuits at the tumor margin [26]. In contrast, CELF2-deficient cells display a mesenchymal and pro-angiogenic gene expression profile, including upregulation of integrin signaling pathways.

Importantly, the CO + EO model revealed that only CELF2-positive GBM cells alter tight junction-associated CLDN5 protein expression at sites of direct contact with endothelial cells, indicating that the capacity to affect vascular integrity and endothelial organization [42, 43] is restricted to specific GBM subpopulations. Collectively, these findings indicate that CELF2 promotes a neural progenitor-like identity and directs GBM cell localization toward neural tissue, whereas CELF2 loss reshapes tumor–microenvironment interactions, enhancing endothelial tropism while promoting a mesenchymal shift. In this context, modulation of CELF2 expression influences both GBM cell phenotype and interactions with the surrounding microenvironment.

To further dissect the molecular basis of the CELF2-dependent endothelial tropism, we identified the scaffold protein AHNAK as a key effector selectively enriched in CELF2-deficient GBM cells. Confocal imaging of CO + EO assembloids revealed co-localization of AHNAK with CD31+ endothelial structures, indicating close spatial proximity and potential physical interactions between CELF2-deficient GBM cells and endothelial cells. Functional silencing of AHNAK in CELF2-deficient cells partially reduced their capacity to infiltrate endothelial regions, confirming its essential role in mediating vascular affinity. Notably, AHNAK is known to associate with tight junction proteins such as CLDN5, ZO-1, and OCLN at the BBB [44], consistent with a role in facilitating endothelial interactions without directly disrupting BBB integrity. Furthermore, high AHNAK expression levels are consistent with its reported barrier-stabilizing properties and its association with reduced tumor growth and improved clinical outcomes, whereas its downregulation correlates with poor prognosis [40], reinforcing its link to a less aggressive tumor phenotype.

### General conclusion

We present a human iPSC-derived cortico-endothelial assembloid model that recapitulates GBM intra-tumoral heterogeneity at the neuronal–vascular interface. This system reveals CELF2 as a key regulator of GBM stem cell tropism, driving either neuroinvasive, aggressive behavior or vascular-associated, less aggressive phenotypes. CELF2-deficient cells adopt a mesenchymal identity and rely on AHNAK for vascular interaction. Our CO + EO assembloid model provides a physiologically relevant platform to study GBM cell plasticity, invasion, and microenvironmental crosstalk, enabling the identification of therapeutic targets that disrupt tissue-specific tumor infiltration.

## Supplementary information

supplemental materials and methods

supplemental figure 1

supplemental figure 2

supplemental figure 3

supplemental figure 4

supplemental figure 5

supplemental figure 6

supplemental figure 7

revised supplemental figure legends

supplemental table 4

QPCR bilan diff mesoderm-vor_original data

QPCR 36B4_original data

QPCR FLT1_original data

QPCR vecadh_original data

QPCR NRP1_original data

blots_original data

supplemental data set table 3

## Data Availability

The data that support the findings of this study are available from the corresponding author upon request.
