# Supplementary material for: Human cortico-vascular assembloids reveal a CELF2-AHNAK-dependent switch from neuronal to endothelial tropism in glioblastoma cells
Source: Cell Death Dis. 2026 Aug 5;17(1):930. doi: 10.1038/s41419-026-09169-z (PMC13728187; doi:10.1038/s41419-026-09169-z)
Supplement: Supplementary file 1 [file 41419_2026_9169_MOESM10_ESM.pdf]

[regulation of synaptic vesicle clustering]

HUMAN|HGNC=1759|UniProtKB=P19022	CDH2	Cadherin-2;CDH2;PTN002513213;orthologs
HUMAN|HGNC=14267|UniProtKB=O14917	PCDH17	Protocadherin-17;PCDH17;PTN002513380;orthologs
HUMAN|HGNC=14290|UniProtKB=Q8NFZ4	NLGN2	Neuroligin-2;NLGN2;PTN002537645;orthologs
HUMAN|HGNC=18994|UniProtKB=Q8TDC3	BRSK1	Serine_threonine-protein kinase BRSK1;BRSK1;PTN002519147;orthologs
HUMAN|HGNC=11405|UniProtKB=Q8IWQ3	BRSK2	Serine_threonine-protein kinase BRSK2;BRSK2;PTN002519146;orthologs

[postsynaptic membrane assembly]

HUMAN|HGNC=8008|UniProtKB=Q9ULB1	NRXN1	Neurexin-1;NRXN1;PTN000013699;orthologs
HUMAN|HGNC=1759|UniProtKB=P19022	CDH2	Cadherin-2;CDH2;PTN002513213;orthologs
HUMAN|HGNC=6696|UniProtKB=O75096	LRP4	Low-density lipoprotein receptor-related protein 4;LRP4;PTN002541524;orthologs
HUMAN|HGNC=14289|UniProtKB=Q9NZ94	NLGN3	Neuroligin-3;NLGN3;PTN002537647;orthologs
HUMAN|HGNC=14290|UniProtKB=Q8NFZ4	NLGN2	Neuroligin-2;NLGN2;PTN002537645;orthologs
HUMAN|HGNC=8009|UniProtKB=Q9P2S2	NRXN2	Neurexin-2;NRXN2;PTN002546086;orthologs

[postsynaptic membrane organization]

HUMAN|HGNC=8008|UniProtKB=Q9ULB1	NRXN1	Neurexin-1;NRXN1;PTN000013699;orthologs
HUMAN|HGNC=29568|UniProtKB=Q7Z7J7	LHFPL4	LHFPL tetraspan subfamily member 4 protein;LHFPL4;PTN002488368;orthologs
HUMAN|HGNC=4232|UniProtKB=P39905	GDNF	Glial cell line-derived neurotrophic factor;GDNF;PTN002486528;orthologs
HUMAN|HGNC=1961|UniProtKB=P11230	AC113189	Acetylcholine receptor subunit beta;CHRNB1;PTN002498468;orthologs
HUMAN|HGNC=1759|UniProtKB=P19022	CDH2	Cadherin-2;CDH2;PTN002513213;orthologs
HUMAN|HGNC=15465|UniProtKB=Q9NQX3	GPHN	Gephyrin;GPHN;PTN002468889;orthologs
HUMAN|HGNC=29861|UniProtKB=Q9BU40	CHRDL1	Chordin-like protein 1;CHRDL1;PTN002478060;orthologs
HUMAN|HGNC=6696|UniProtKB=O75096	LRP4	Low-density lipoprotein receptor-related protein 4;LRP4;PTN002541524;orthologs
HUMAN|HGNC=14289|UniProtKB=Q9NZ94	NLGN3	Neuroligin-3;NLGN3;PTN002537647;orthologs
HUMAN|HGNC=35409|UniProtKB=A6NL88	SHISA7	Protein shisa-7;SHISA7;PTN002527865;orthologs
HUMAN|HGNC=14290|UniProtKB=Q8NFZ4	NLGN2	Neuroligin-2;NLGN2;PTN002537645;orthologs
HUMAN|HGNC=613|UniProtKB=P02649	APOE	Apolipoprotein E;APOE;PTN002498710;orthologs
HUMAN|HGNC=14294|UniProtKB=Q9BYB0	SHANK3	SH3 and multiple ankyrin repeat domains protein 3;SHANK3;PTN002516415;orthologs
HUMAN|HGNC=8009|UniProtKB=Q9P2S2	NRXN2	Neurexin-2;NRXN2;PTN002546086;orthologs

[synapse maturation]

HUMAN|HGNC=15474|UniProtKB=Q9Y566	SHANK1	SH3 and multiple ankyrin repeat domains protein 1;SHANK1;PTN002516409;orthologs
HUMAN|HGNC=8008|UniProtKB=Q9ULB1	NRXN1	Neurexin-1;NRXN1;PTN000013699;orthologs
HUMAN|HGNC=10763|UniProtKB=Q9BYH1	SEZ6L	Seizure 6-like protein;SEZ6L;PTN002499804;orthologs
HUMAN|HGNC=23059|UniProtKB=Q96GW7	BCAN	Brevican core protein;BCAN;PTN002504038;orthologs
HUMAN|HGNC=8594|UniProtKB=O75781	PALM	Paralemmin-1;PALM;PTN002471088;orthologs
HUMAN|HGNC=6657|UniProtKB=Q8TBB1	LNX1	E3 ubiquitin-protein ligase LNX;LNX1;PTN002500752;orthologs
HUMAN|HGNC=29861|UniProtKB=Q9BU40	CHRDL1	Chordin-like protein 1;CHRDL1;PTN002478060;orthologs
HUMAN|HGNC=30844|UniProtKB=Q6UXD5	SEZ6L2	Seizure 6-like protein 2;SEZ6L2;PTN002499810;orthologs

HUMAN|HGNC=28246|UniProtKB=Q96ID5	IGSF21	Immunoglobulin superfamily member 21;IGSF21;PTN002551519;orthologs
HUMAN|HGNC=26011|UniProtKB=Q9NX95	SYBU	Syntabulin;SYBU;PTN002497220;orthologs
HUMAN|HGNC=15955|UniProtKB=Q53EL9	SEZ6	Seizure protein 6 homolog;SEZ6;PTN002499802;orthologs

presynaptic membrane assembly

HUMAN|HGNC=8008|UniProtKB=Q9ULB1	NRXN1	Neurexin-1;NRXN1;PTN000013699;orthologs
HUMAN|HGNC=6696|UniProtKB=O75096	LRP4	Low-density lipoprotein receptor-related protein 4;LRP4;PTN002541524;orthologs
HUMAN|HGNC=14289|UniProtKB=Q9NZ94	NLGN3	Neuroligin-3;NLGN3;PTN002537647;orthologs
HUMAN|HGNC=14287|UniProtKB=Q8N0W4	NLGN4X	Neuroligin-4, X-linked;NLGN4X;PTN002537636;orthologs
HUMAN|HGNC=9668|UniProtKB=P23468	PTPRD	Receptor-type tyrosine-protein phosphatase delta;PTPRD;PTN002498779;orthologs
HUMAN|HGNC=14290|UniProtKB=Q8NFZ4	NLGN2	Neuroligin-2;NLGN2;PTN002537645;orthologs

excitatory chemical synaptic transmission

HUMAN|HGNC=4588|UniProtKB=O15399	GRIN2D	Glutamate receptor ionotropic, NMDA 2D;GRIN2D;PTN002540160;orthologs
HUMAN|HGNC=4586|UniProtKB=Q13224	GRIN2B	Glutamate receptor ionotropic, NMDA 2B;GRIN2B;PTN002540152;orthologs
HUMAN|HGNC=4585|UniProtKB=Q12879	GRIN2A	Glutamate receptor ionotropic, NMDA 2A;GRIN2A;PTN002540171;orthologs
HUMAN|HGNC=29861|UniProtKB=Q9BU40	CHRDL1	Chordin-like protein 1;CHRDL1;PTN002478060;orthologs
HUMAN|HGNC=4587|UniProtKB=Q14957	GRIN2C	Glutamate receptor ionotropic, NMDA 2C;GRIN2C;PTN002540163;orthologs
HUMAN|HGNC=6297|UniProtKB=O43525	KCNQ3	Potassium voltage-gated channel subfamily KQT member 3;KCNQ3;PTN002479939;orthologs
HUMAN|HGNC=34491|UniProtKB=Q6ZSJ9	SHISA6	Protein shisa-6;SHISA6;PTN002527868;orthologs

synaptic vesicle clustering

HUMAN|HGNC=8008|UniProtKB=Q9ULB1	NRXN1	Neurexin-1;NRXN1;PTN000013699;orthologs
HUMAN|HGNC=11494|UniProtKB=P17600	SYN1	Synapsin-1;SYN1;PTN002474386;orthologs
HUMAN|HGNC=9777|UniProtKB=P20336	RAB3A	Ras-related protein Rab-3A;RAB3A;PTN002514971;orthologs
HUMAN|HGNC=1759|UniProtKB=P19022	CDH2	Cadherin-2;CDH2;PTN002513213;orthologs
HUMAN|HGNC=11496|UniProtKB=O14994	SYN3	Synapsin-3;SYN3;PTN002474387;orthologs
HUMAN|HGNC=15885|UniProtKB=Q9H7V2	SYNDIG1	Synapse differentiation-inducing gene protein 1;SYNDIG1;PTN002495110;orthologs
HUMAN|HGNC=1117|UniProtKB=Q9UPA5	BSN	Protein bassoon;BSN;PTN002493831;orthologs

regulation of synaptic plasticity

HUMAN|HGNC=11371|UniProtKB=Q9NUL3	STAU2	Double-stranded RNA-binding protein Staufen homolog 2;STAU2;PTN002474961;orthologs
HUMAN|HGNC=30500|UniProtKB=Q7Z6L0	PRRT2	Proline-rich transmembrane protein 2;PRRT2;PTN002495231;orthologs
HUMAN|HGNC=1004|UniProtKB=Q4VC05	BCL7A	B-cell CLL_lymphoma 7 protein family member A;BCL7A;PTN002489634;orthologs
HUMAN|HGNC=14203|UniProtKB=Q8WXH2	JPH3	Junctophilin-3;JPH3;PTN002507918;orthologs
HUMAN|HGNC=11953|UniProtKB=Q92752	TNR	Tenascin-R;TNR;PTN002499053;orthologs
HUMAN|HGNC=9390|UniProtKB=P31321	PRKAR1B	cAMP-dependent protein kinase type I-beta regulatory subunit;PRKAR1B;PTN002481194;orthologs
HUMAN|HGNC=3289|UniProtKB=Q13542	EIF4EBP2	Eukaryotic translation initiation factor 4E-binding protein 2;EIF4EBP2;PTN002489291;orthologs
HUMAN|HGNC=1874|UniProtKB=P23528	CFL1	Cofilin-1;CFL1;PTN002484467;orthologs
HUMAN|HGNC=8000|UniProtKB=Q92686	NRGN	Neurogranin;NRGN;;orthologs

HUMAN|HGNC=6231|UniProtKB=Q14721	KCNB1	Potassium voltage-gated channel subfamily B member 1;KCNB1;PTN002479964;orthologs
HUMAN|HGNC=4588|UniProtKB=O15399	GRIN2D	Glutamate receptor ionotropic, NMDA 2D;GRIN2D;PTN002540160;orthologs
HUMAN|HGNC=9777|UniProtKB=P20336	RAB3A	Ras-related protein Rab-3A;RAB3A;PTN002514971;orthologs
HUMAN|HGNC=7761|UniProtKB=O76050	NEURL1	E3 ubiquitin-protein ligase NEURL1;NEURL1;PTN002488146;orthologs
HUMAN|HGNC=7763|UniProtKB=Q15784	NEUROD2	Neurogenic differentiation factor 2;NEUROD2;PTN002499521;orthologs
HUMAN|HGNC=4586|UniProtKB=Q13224	GRIN2B	Glutamate receptor ionotropic, NMDA 2B;GRIN2B;PTN002540152;orthologs
HUMAN|HGNC=18351|UniProtKB=B8ZZ34	SHISA8	Protein shisa-8;SHISA8;PTN002527873;orthologs
HUMAN|HGNC=19409|UniProtKB=O43300	LRRTM2	Leucine-rich repeat transmembrane neuronal protein 2;LRRTM2;PTN002540731;orthologs
HUMAN|HGNC=1449|UniProtKB=P0DP25	CALM3	Calmodulin-3;CALM3;PTN004596978;orthologs
HUMAN|HGNC=4585|UniProtKB=Q12879	GRIN2A	Glutamate receptor ionotropic, NMDA 2A;GRIN2A;PTN002540171;orthologs
HUMAN|HGNC=4597|UniProtKB=P41594	GRM5	Metabotropic glutamate receptor 5;GRM5;PTN002514248;orthologs
HUMAN|HGNC=29861|UniProtKB=Q9BU40	CHRDL1	Chordin-like protein 1;CHRDL1;PTN002478060;orthologs
HUMAN|HGNC=13447|UniProtKB=Q9H2H9	SAT1	Sodium-coupled neutral amino acid symporter 1;SLC38A1;PTN002505919;orthologs
HUMAN|HGNC=4594|UniProtKB=Q14416	GRM2	Metabotropic glutamate receptor 2;GRM2;PTN002514253;orthologs
HUMAN|HGNC=12703|UniProtKB=O76090	BEST1	Bestrophin-1;BEST1;PTN002473285;orthologs
HUMAN|HGNC=10500|UniProtKB=P04271	S100B	Protein S100-B;S100B;PTN002481294;orthologs
HUMAN|HGNC=5182|UniProtKB=P35367	HRH1	Histamine H1 receptor;HRH1;PTN002538164;orthologs
HUMAN|HGNC=11498|UniProtKB=O43759	SYNGR1	Synaptogyrin-1;SYNGR1;PTN002474384;orthologs
HUMAN|HGNC=2310|UniProtKB=Q6PUV4	CPLX2	Complexin-2;CPLX2;PTN002497634;orthologs
HUMAN|HGNC=27012|UniProtKB=Q6P5X5	C22orf39	UPF0545 protein C22orf39;C22orf39;PTN002523785;orthologs
HUMAN|HGNC=9630|UniProtKB=P21246	PTN	Pleiotrophin;PTN;PTN002493158;orthologs

regulation of long-term synaptic depression

HUMAN|HGNC=11371|UniProtKB=Q9NUL3	STAU2	Double-stranded RNA-binding protein Staufen homolog 2;STAU2;PTN002474961;orthologs
HUMAN|HGNC=6231|UniProtKB=Q14721	KCNB1	Potassium voltage-gated channel subfamily B member 1;KCNB1;PTN002479964;orthologs
HUMAN|HGNC=4597|UniProtKB=P41594	GRM5	Metabotropic glutamate receptor 5;GRM5;PTN002514248;orthologs
HUMAN|HGNC=6893|UniProtKB=P10636	MAPT	Microtubule-associated protein tau;MAPT;PTN002479589;orthologs
HUMAN|HGNC=4576|UniProtKB=O43424	GRID2	Glutamate receptor ionotropic, delta-2;GRID2;PTN002498660;orthologs
HUMAN|HGNC=262|UniProtKB=P30542	ADORA1	Adenosine receptor A1;ADORA1;PTN002517551;orthologs
HUMAN|HGNC=320|UniProtKB=Q15109	AGER	Advanced glycosylation end product-specific receptor;AGER;PTN002485496;orthologs
HUMAN|HGNC=648|UniProtKB=Q7LC44	ARC	Activity-regulated cytoskeleton-associated protein;ARC;PTN002496804;orthologs
HUMAN|HGNC=14294|UniProtKB=Q9BYB0	SHANK3	SH3 and multiple ankyrin repeat domains protein 3;SHANK3;PTN002516415;orthologs
