# Supplementary material for: Human cortico-vascular assembloids reveal a CELF2-AHNAK-dependent switch from neuronal to endothelial tropism in glioblastoma cells
Source: Cell Death Dis. 2026 Aug 5;17(1):930. doi: 10.1038/s41419-026-09169-z (PMC13728187; doi:10.1038/s41419-026-09169-z)
Supplement: Supplementary file 3 [file 41419_2026_9169_MOESM9_ESM.pdf]

Supplemental figure legends

Suppl. Figure 1. Efficient *in vitro* induction of mesodermal tissue and endothelial organoids. A,B) Endothelial organoids (EOs) were derived from hiPSCs (A) *via* embryoid body formation and subsequent culture in VEGF/FGF2-containing medium, resulting in the emergence of elongated, multicellular structures reminiscent of blood vessels (B). **C)** Real-time qRT-PCR analysis of mesodermal and endothelial markers showing robust mesodermal induction (BRACHYURY, BRY) followed by endothelial maturation, as indicated by upregulation of VEGFR1, VEGFR2, and NRP1. **D–F)** Immunostaining of *in vitro*–differentiated endothelial cells in EOs showing expression of VE-CADHERIN (green, D and E) and CD31 (red in E; green in F), with no detectable expression of the neuronal marker MAP2 (red in F). **G)** Western blot analysis of PDGFRβ expression in two distinct EO batches, showing detectable levels indicative of the presence of pericytes. **H)** Immunostaining of CD31+ endothelial cells (red) and PDGFRβ+ pericytes (green), indicated by arrowheads in the high-magnification images. **I)** Light-sheet microscopy orthogonal plane scans of endothelial cell structures in CO+EOs labeled for CD31 (white), revealing luminal structures reminiscent of rudimentary blood vessel lumens (arrowheads). **J)** CD31 (red) and GFAP (white) immunostaining showing GFAP+ astrocytes (arrowheads) accumulating in endothelial-enriched areas of CO+EOs. Scale bars: 100 µm.

Suppl. Figure 2. Short hairpin-mediated CELF2 knock-down in patient-derived GBM cells. A) Western blot analysis of CELF2 (left panel) and TUBB1 (right panel) expression in patient-derived GBM cells (Patient) and in two cultured GBM cell lines (GB5 and TG6) expressing either an anti-CELF2 short hairpin RNA (shCELF2) or a scrambled control sequence (shCTRL). High levels of CELF2 detected in patient-derived samples and in shCTRL GBM cells were markedly reduced in the shCELF2 condition, confirming efficient CELF2 knockdown. **B)** Immunostaining for CELF2 (red) and GFP (green) in shCTRL (upper row) and shCELF2 (lower row) GB5 cells one week after co-culture with CO+EOs, confirming stable CELF2 modulation in the co-culture setup. Scale bars: 100 µm.

Suppl. Figure 3. CELF2-dependent distribution and stemness identity of GBM cells in CO+EOs. A,B) TUJ1 (red) and GFP (green) immunostaining of CO+EOs with automated classification of tumor cells (green), cortical domains (red), and endothelial domains (yellow); graphs in B show the percentage of each surface/cell type in human assembloids 3 weeks after GBM invasion: tumor-free MAP2+ cortical surface (left), tumor-free endothelial surface (middle), and GFP+ GBM cells (right). **C,D)** Automated recognition of tumor (green), endothelial (yellow), and cortical (red) surfaces in CO+EOs 3 weeks after invasion with TG6 shCTRL or TG6 shCELF2 cells, showing the same tropism toward mesodermal tissue upon CELF2 downregulation as found with GB5 cells. Graphs in D show the percentage of GFP+ cells found in the CO or EO domains. **E,F)** Triple immunostaining for GFP (green), KI67 (red), and OLIG2 (blue) in shCTRL and shCELF2 assembloids after 3 weeks of co-culture (E). Graphs in F show the percentages of GFP+ cells positive for KI67, OLIG2, or both KI67 and OLIG2, indicating marked loss of OLIG2+ GSC identity upon CELF2 downregulation. Scale bars: 100 µm.

Suppl. Figure 4. Glioblastoma-invaded COs reveal CELF2-dependent dynamics of GBM cell morphology, migration, proliferation, and stemness. A) Brightfield images showing COs one week after co-culture with shCTRL GB5 cells (top row) or with shCELF2 GB5 cells (lower row); CELF2-deprived cells can be seen on the organoid surface and with round morphology. **B)** ImageJ quantification of cellular morphology (circularity and roundness) of GFP+ shCTRL and shCELF2 GB5 cells after one week of co-culture in COs. **C)** 3D reconstruction of tissue-cleared COs invaded by shCTRL or shCELF2 GB5 cells, as indicated; automated cell recognition in Imaris allowed measurement of the distance of cells (dots) from the organoid surface; blue to red color code indicates far to close to the surface, respectively. **D,E)** Triple immunostaining for GFP (green), KI67 (red), and OLIG2 (blue) in shCTRL and shCELF2-supplemented COs after three weeks. The percentages of GFP+ cells positive for KI67, OLIG2, or both KI67 and OLIG2 are shown in graph E. Scale bars: 100 µm.

Suppl. Figure 5. CELF2-depleted TG6 GBM cells show impaired migratory potential in COs and increased invasiveness in EOs. A-E) MAP2 (red) and GFP (green) immunostaining of day 111 cortical organoids (COs) after one week of co-culture (A) or after three weeks of co-culture (B) with TG6 control or CELF2-deprived cells. Quantification of GFP+ tumoral surface area after one week or after three weeks of co-culture is shown in C and D, respectively. Graph E displays the position of GBM cells within organoid sections (on the surface, in the 100 µm radius peripheral outer shell, or in the inner core) for TG6 shCTRL and shCELF2 COs after one week of co-culture. **F)** CD31 (yellow; endothelial cells)

and GFP (green; tumoral cells) immunostaining of EOs after 3 weeks of co-culture with GBM cells with TG6 shCTRL and shCELF2 cells. As for GB5 cells, CELF2-depleted TG6 cells exhibit tropism toward endothelial tissues and demonstrate high invasive potential into CD31+ domains. Arrowheads indicate clusters of GFP+ cells. Scale bars: 100 µm.

Suppl. Figure 6. Varying degrees of vascular enrichment in mitotic *versus* non-mitotic zones in GBM patient samples and CELF2+ cell distribution. A,B) Immunohistochemical staining of GBM samples showing the mitotic marker MIB-1 (brown in panel A) and the astrocyte differentiation marker GFAP (brown in panel B), overlaid on hematoxylin-eosin (H&E) staining, which highlights nuclei in violet. The upper low-magnification images illustrate intra-tumoral heterogeneity, with mitotic regions (Zone A) displaying sparse vasculature, and non-mitotic regions (Zone B) showing abnormal, dense vasculature (v) adjacent to necrotic areas (n). High-magnification images reveal strong MIB-1 labeling in mitotic zones and high GFAP labeling in non-mitotic zones. Scale bars: 800 µm (low magnification) and 200 µm (high magnification). **C-E)** Immunohistochemical staining of GBM samples from three patients showing CELF2 (brown) with hematoxylin counterstaining highlighting nuclei (violet). The upper row displays areas with few and small blood vessels (normal vascular domains), whereas the lower row shows regions enriched in large, abnormal blood vessels (abnormal, vessel-enriched domains). Quantification of CELF2+ cells across these domains is presented in Figure 4. Scale bars: 100 µm.

Suppl. Figure 7. AHNAK expression in both GBM and endothelial cells in close spatial proximity in CO+EOs. Immunostaining for CD31 (red; endothelial cells), GFP (green; TG6 shCELF2 cells), and AHNAK (white) in day 110 CO+EOs after 3 weeks of co-culture with GBM cells. Low AHNAK expression is detected in CD31+ endothelial cells (arrowheads), whereas stronger AHNAK staining is observed in GFP+ GBM cells (dotted arrowheads) localized adjacent to endothelial domains. Scale bars: 100 µm.
